# Co-occurrence or dependence? Using spatial analyses to explore the interaction between palms and *Rhodnius* triatomines

**DOI:** 10.1186/s13071-020-04088-0

**Published:** 2020-04-22

**Authors:** Johan M. Calderón, Camila González

**Affiliations:** Centro de Investigaciones en Microbiología y Parasitología Tropical, (CIMPAT), Departamento de Ciencias Biológicas, Universidad de Los Andes, Bogotá D.C., Colombia

**Keywords:** Triatomines, *Rhodnius*-infested palms, Ecological niche modeling, Niche similarity, Unlinked biotic predictors

## Abstract

**Background:**

Triatomine bugs are responsible for the vectorial transmission of the parasite *Trypanosoma cruzi*, the etiological agent of Chagas disease, a zoonosis affecting 10 million people and with 25 million at risk of infection. Triatomines are associated with particular habitats that offer shelter and food. Several triatomine species of the genus *Rhodnius* have a close association with palm crowns, where bugs can obtain microclimatic stability and blood from the associated fauna. The *Rhodnius*-palm interaction has been reported in several places of Central and South America. However, the association in the distributions of *Rhodnius* species and palms has not been explicitly determined.

**Methods:**

Niches of *Rhodnius* and palm species with reports of *Rhodnius* spp. infestation were estimated by minimum volume ellipsoids and compared in the environmental and the geographical space to identify niche similarity. *Rhodnius* spp. niche models were run with the palm distributions as environmental variables to determine if palm presence could be considered a predictor of *Rhodnius* spp. distributions, improving model performance.

**Results:**

Niche similarity was found between all the studied *Rhodnius* and palm species showing variation in niche overlap among the involved species. Most of the areas with suitable conditions for *Rhodnius* species were also suitable to palm species, being favorable for more than one palm species in the majority of locations. Performance was similar in *Rhodnius* niche models with and without palm distributions. However, when palm distributions were included, their contribution to the model was high, being the most important variable in some *Rhodnius* spp.

**Conclusions:**

To our knowledge, this is the first time that the distributions of *Rhodnius* and palm species were compared on a large scale and their spatial association explicitly studied. We found spatial association between *Rhodnius* and palm species can be explained because both organisms shared environmental requirements, and most of the areas with suitable conditions for *Rhodnius* species were also suitable to several palm species. *Rhodnius* presence would not be restricted to palm presence but the zones with palm presence could be more suitable for *Rhodnius* spp. presence. 
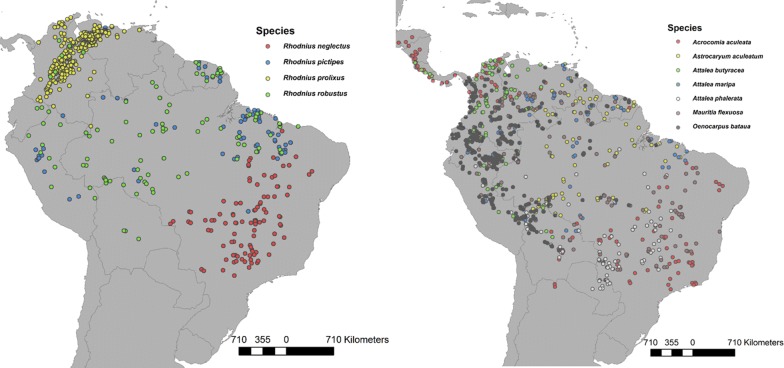

## Background

Triatomine bugs are responsible for the vectorial transmission of the parasite *Trypanosoma cruzi*, the etiological agent of Chagas disease, a zoonosis affecting 10 million people and with 25 million at risk of infection [1]. Triatomines show associations with particular habitats that offer shelter and food [2]; this association can be specific to one type of habitat, as occurs with *Psammolestes* triatomines living in bird nests, or to several habitats such as *Triatoma sordida*, which can be found in rock piles, hollow trees and human dwellings [3]. Several species belonging to the genus *Rhodnius* have been found in close association with palms in their sylvatic cycle [4], some related to a particular type of palm such as *Rhodnius brethesi* to *Leopoldinia piassaba* palms, and others, such as *R. robustus*, associated with several palm species [5]. Palm crowns have been suggested as suitable habitats for *Rhodnius* bugs due to their inner microclimatic stability and food availability. Microclimatic stability of the palm is likely to be the result of leaf insertion, creating a highly protected environment with a stable temperature and humidity [6], while blood sources for the *Rhodnius* bugs, are provided by the fauna visiting or inhabiting the palm [6]. The fact that palms are inhabited by Chagas disease vectors is important from a public health perspective, since insects living in palms can infest nearby houses [7]. The migration of *Rhodnius* vectors from palms to households could threaten vector control programs conducted during Chagas disease control initiatives, since re-infestation of insecticide-treated dwellings can occur [8].

In the Americas, palms are distributed from southern USA to northern Argentina and central Chile [9]. From 550 palm species naturally occurring in the Americas, 19 have been reported to be infested by *Rhodnius* bugs (Additional file 1: Table S1). *Rhodnius* species are distributed from Central America to Bolivia, with the Amazon region the zone showing the greatest number of species [10]. Some *Rhodnius* species, such as *R. pictipes* and *R. robustus*, have very wide geographical distributions including several countries; while others, such as *R. ecuadoriensis* and *R. colombiensis*, are restricted to certain regions inside one or two countries [11]. Palms infested by *Rhodnius* bugs have been observed and reported in numerous areas in Central and South America [5] suggesting that sylvatic *Rhodnius* spp. distributions broadly coincide with palm distributions [2]. However, the role of palm presence as a determinant variable on *Rhodnius* spp. distributions has not been explicitly assessed.

Under normal reproduction and dispersal conditions, a species is predicted to be present in a geographical region that is directly congruent with the distribution of its Grinnellian niche [12]. This niche interpretation focuses on conditions necessary for the species’ existence, and it has been extensively used in studies of niche estimation and species distribution analyses [13, 14]. *Rhodnius* and palm species would occupy similar geographical regions if their Grinnellian niches were similar. Moreover, the locations with suitable conditions for both *Rhodnius* and palm species could be considered as potential zones of *Rhodnius*-palm co-occurrence.

The aim of this study was to determine if there is a close spatial and ecological association on a broad scale, between *Rhodnius* spp. and *Rhodnius*-infested palms, suggesting habitat dependence. To do so, the similarity between Grinnellian niches of *Rhodnius* spp. and infested palm species was determined in both the environmental and the geographical space. Additionally, the role of palm presence as an important predictor of *Rhodnius* spp. distributions was evaluated through the use of ecological niche models (ENMs).

## Methods

### Database assemblage

Species occurrences (i.e. geographical coordinates from where the species had been collected) were obtained for *Rhodnius* species (1980–2000) from “DataTri” [15], and for palms from the Global Biodiversity Information Facility (GBIF) (1980–2000), downloaded in October 2018 using the “gbif” function of the *dismo* R package [16] (*Rhodnius* and palm species are listed in Additional file 1: Table S1). *Rhodnius* spp. occurrences in “DataTri” include records from domestic, peridomestic and sylvatic habitats; however not all records have this information, therefore origin could not be used as a filter [15]. The databases were depurated by choosing only georeferenced occurrences, removing duplicated records, and validated with known geographical distributions reported in the literature [17–20]. Also, occurrences in elevations outside species limits were omitted for both *Rhodnius* spp. and palms [9, 11, 17]. *Rhodnius prolixus* occurrences in Central America were excluded from the study since they have never been associated with palms [21], and *R. prolixus* are no longer found in previously reported areas of Central America as a possible consequence of vector control initiatives.

To reduce the effect of sampling bias in the occurrence dataset, spatial thinning was performed with the *spThin* R package [22] using a minimum nearest neighbor distance greater than or equal to 10 km. This distance was chosen based on the high spatial heterogeneity, and the same distance has been used in previous studies on highly heterogeneous areas [23, 24]. Moreover, this distance is much larger than the flight dispersal reported for some *Rhodnius* species (e.g. *c.*200 m for *R. prolixus*) [25], avoiding the use of many occurrences from closely located populations.

### Niche estimation and comparison

To identify niche similarity between *Rhodnius* and palms species, their Grinnellian niches were estimated and compared in the environmental and geographical space. For this purpose, an initial set of environmental variables was composed including the 19 bioclimatic variables from WorldClim [26], and 42 variables with remote sensing information of land surface temperature (LST), normalized difference vegetation index (NDVI) and middle infrared radiation (MIR). The remote sensing variables were calculated from AVHRR (advanced very high-resolution radiometer) images and processed by the TALA group (Oxford University, UK) using the temporal decomposition of Fourier [27]. Pearsonʼs correlation coefficient was calculated among environmental variables to avoid collinearity, and when a group of variables with high correlation was found (i.e. absolute *r*-value > 0.7), only one variable was selected. This selection was based on which variable grouped more temporal information (e.g. yearly over monthly). The final nine selected environmental variables were five bioclimatic variables (BIO 1, annual mean temperature; BIO 2, mean diurnal range; BIO 12, annual precipitation; BIO 15, precipitation seasonality; and BIO 18, precipitation of warmest quarter), and four remote sensing variables (mean LST, LST annual phase, mean NDVI, and NDVI annual phase). Correlation was double-checked by the variable inflation factor, obtaining values lower than three for each variable. The spatial resolution of all the environmental layers was 2.5° (~8 km^2^).

Only *Rhodnius* and palm species with more than 90 occurrences were considered for niche analyses. This threshold was determined by the number of environmental variables used, following the suggestion of Guisan et al. [28] of at least 10 records per environmental variable.

Grinnellian niches were estimated as minimum-volume ellipsoids (MVE) in the environmental space using the NicheA software v. 3.0 [29]. As background data, the three first PCAs from the nine environmental variables were obtained (72% of total variation). The background extent included the continental Neotropics from southern Nicaragua to Bolivia [15], the area corresponding to *Rhodnius* spp. distributional range including five degrees below and above the latitudinal known limits. Overlaps between each *Rhodnius* and palm species MVEs were estimated using NicheA (with a default precision of 0.01). Along with the niches of *Rhodnius* and palm species, we estimated a niche for the genus *Rhodnius* and another considering all the infested palms studied here. The *Rhodnius* niche was estimated with occurrences of all *Rhodnius* species, and the infested palms niche with the occurrences of all palm species infested by any *Rhodnius* species (species in Additional file 1: Table S1). To allow comparisons between *Rhodnius* species, niche overlaps were normalized by the *Rhodnius* species niche volume (= (Niche overlap volume/MVEs volume of the *Rhodnius* species) × 100).

Palms and *Rhodnius* MVEs were projected and compared on the geographical space and used to estimate the potential areas of *Rhodnius* spp. and palm co-occurrence. For each *Rhodnius* and palm species, 80% of the occurrences located within the MVEs were randomly drawn using the “probability” method (NicheA converted the probability according to a logistic function of threshold *β* = 0.7 and slope *α* = -0.05, and sampled based on the converted probability. The areas with high probability contain more occurrences). With those occurrences, ENMs were obtained using the maximum entropy algorithm (MaxEnt v. 3.4.1) [30] (with 10,000 background points, 500 iterations, regularization coefficient = 1, linear, quadratic and product feature classes, and log-log output). Binary maps were obtained using the 10% error threshold, and the areas where estimated *Rhodnius* spp. and palms niches overlapped were considered as potential areas for *Rhodnius*-palm co-occurrence. Niches comparisons were carried out inside the geographical range of each *Rhodnius* species including five degrees far from the known geographical limits.

### Palm distributions as predictors of *Rhodnius* ENMs

To determine if palm presence could be considered a predictor of *Rhodnius* species distributions, *Rhodnius* models were run twice: first with only environmental variables (the same nine variables used in the niche estimation), and again including palm distributions as environmental variables (the nine environmental variables plus seven palm niche distributions). Palm distribution could be considered an appropriate predictor for ENMs because it is a variable not affected by the presence of a *Rhodnius* species (i.e. unlinked variable) [31]. For representation of the niche, unlinked environmental variables are preferred [32], because linked variables (i.e. affected by the focal species) can increase the complexity of the niche representation due to possible feedbacks between variables [31]. Palm potential distributions used here were the palm niche geographical projections (continuous outputs) obtained from the previous section.

ENMs were run for each *Rhodnius* species and the calibration area was the species geographical range including five degrees below and above the known limits. Maximum entropy (MaxEnt v. 3.4.1) was used as modeling algorithm (with 10,000 background points 500 iterations, and log-log output). Several regularization coefficients (0.02, 0.1, 0.46, 1, 2.2 and 4.6) and feature classes (linear, quadratic and product) were tested for each species with the *ENMeval* R package [33], and the options giving the lowest corrected Akaike information criterion (AICc) were selected (Additional file 1: Table S2). For each species, ENMs were run ten times with different presence samples to test robustness [28], and model evaluation was performed each time. In each repetition, 80% of the occurrences were randomly chosen for training the model and the remaining 20% of the occurrences used for testing. Two evaluation methods were carried out: partial area under the ROC curve (pAUC) [34] and omission rates [14]. The pAUC with an error of 0.10 and its ratio to the AUC null model were calculated for each repetition (performed with NicheA). Ten-percentile and zero-percentile training omission rates (proportion of testing occurrences omitted with each threshold) were calculated along with their predicted presence area (performed with MaxEnt). Evaluation statistics were compared between *Rhodnius* ENMs with and without palm distributions using a paired t-test (*α* = 0.05). To this purpose, the same training and test datasets were used in both cases.

The final continuous map for each *Rhodnius* species was the mean of the ten obtained outputs (from the repetitions), and the uncertainty map was the standard deviation of those outputs. The continuous map was transformed to a binary map using the mean of the ten-percentile thresholds of the outputs.

## Results

### Database assemblage

**Fig. 1:**
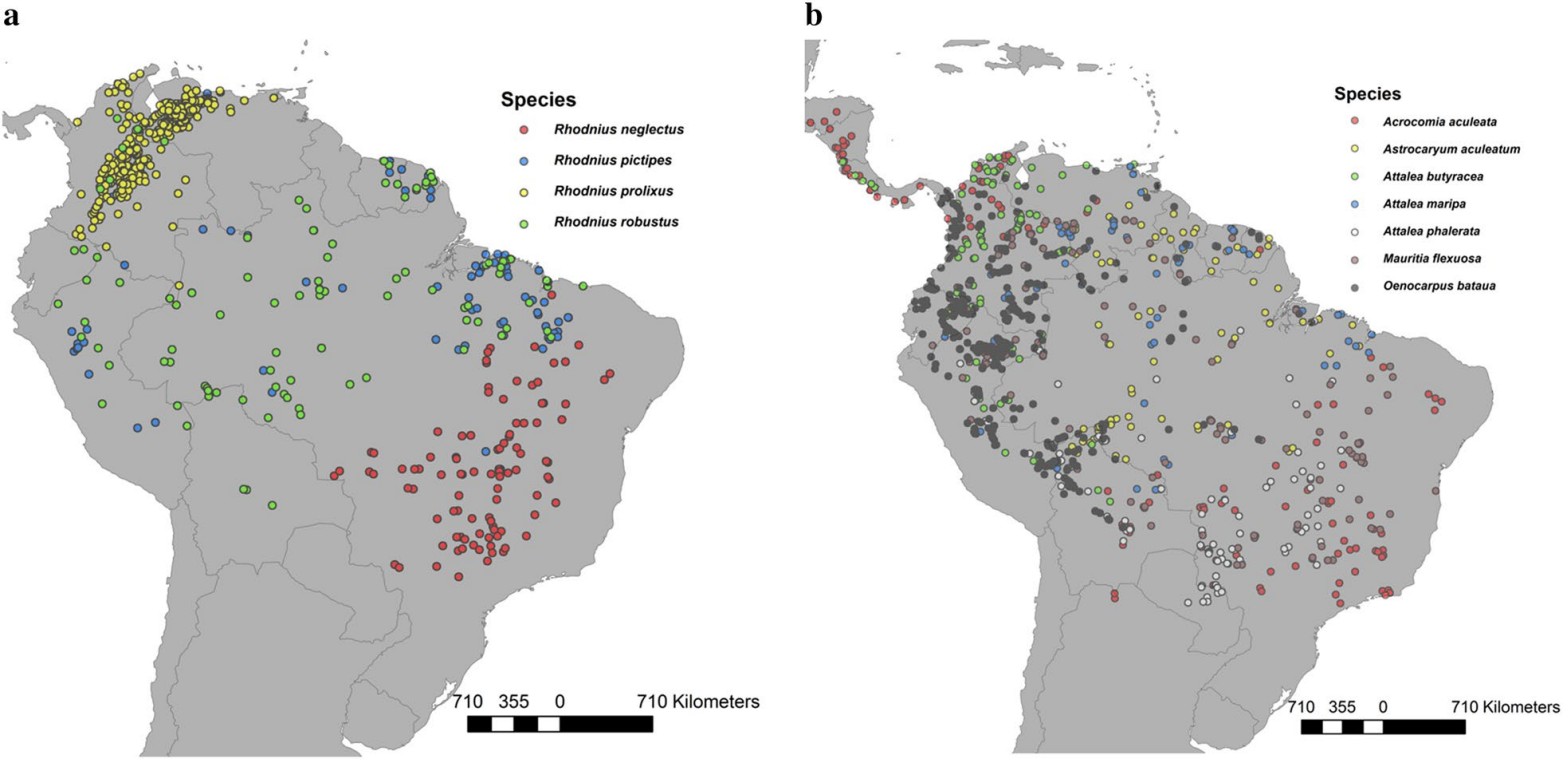
**a** Final set of *Rhodnius* occurrences. **b** Final set of infested palm occurrences. Maps elaborated with ArcGis 10.4.1. Occurrences data obtained from DataTri [15] and GBIF

Inside the background area, we obtained 1930 records of *Rhodnius* species and 5412 records of *Rhodnius*-infested palm species. After depuration and spatial thinning, the final dataset consisted of 930 records of *Rhodnius* species and 1757 of infested palms. Four *Rhodnius* species were selected, which had more than 90 occurrences: *R. neglectus*, *R. pictipes*, *R. prolixus* and *R. robustus.* By the same criteria, seven palm species were selected: *Acrocomia aculeata*, *Astrocaryum aculeatum*, *Attalea butyracea*, *Attalea maripa*, *Attalea phalerata*, *Mauritia flexuosa* and *Oenocarpus bataua* (Fig. 1, Table 1).

**Table 1:** *Rhodnius* and palm occurrences

Organism	Species	Initial dataset	Final dataset^a^
*Rhodnius*	*R. neglectus*	143	102
	*R. pictipes*	166	130
	*R. prolixus*	1215	352
	*R. robustus*	116	96
Palms	*Ac. aculeata*	395	157
	*As. aculeatum*	256	120
	*A. butyracea*	307	155
	*A. maripa*	292	160
	*A. phalerata*	329	150
	*M. flexuosa*	306	206
	*O. bataua*	724	326

^a^Data set obtained after data depuration and spatial thinning

### Niche estimation and comparison

**Fig. 2:**
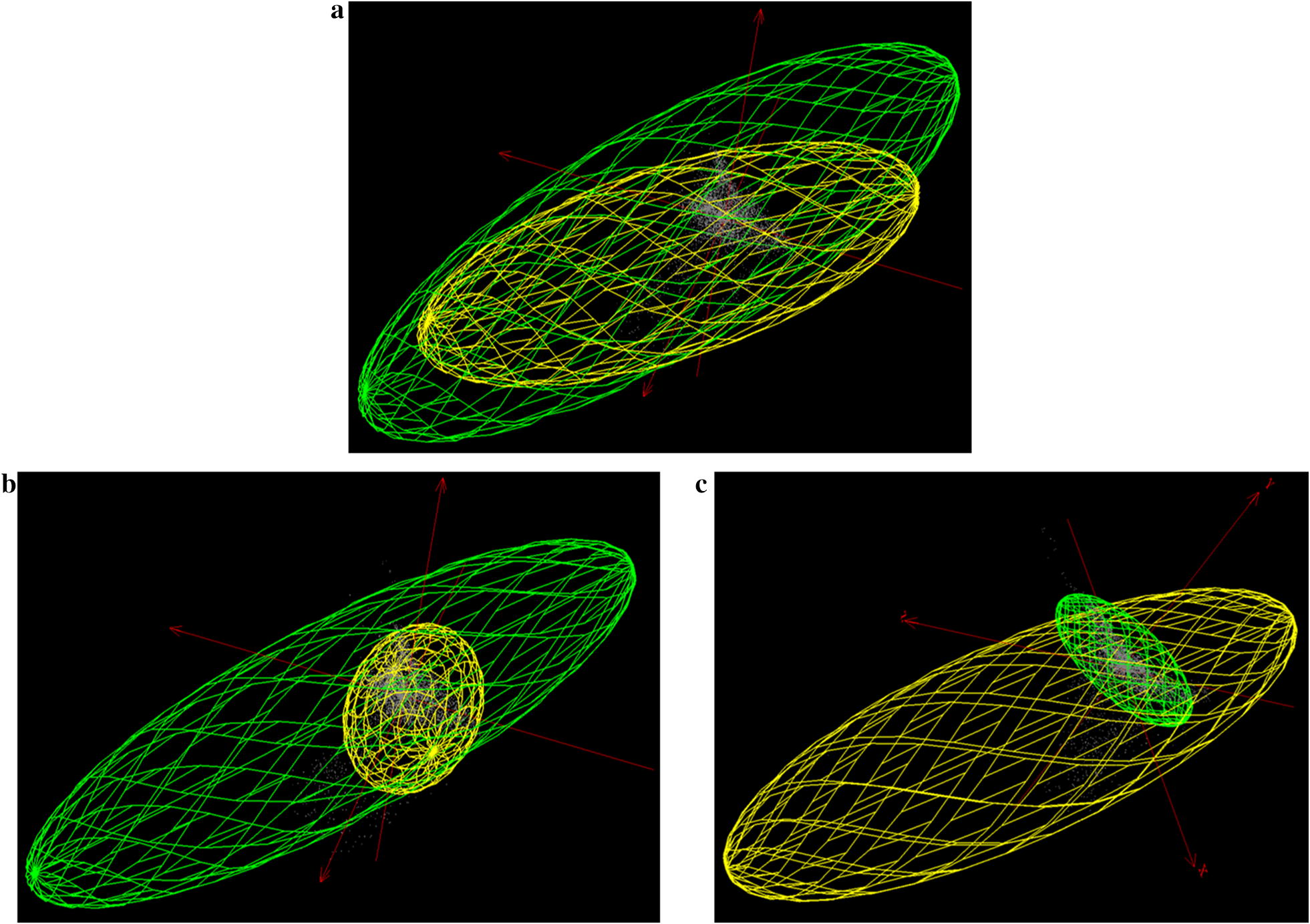
Minimum-volume ellipsoids for *Rhodnius* (yellow) and for palms (green). The niche overlap corresponded to the niche volume shared by both ellipsoids. Gray dots indicate background data. **a** Overlap between the genus *Rhodnius* and infested palm species. **b** Overlap between *R. pictipes* and *M. flexuosa*. **c** Overlap between *R. neglectus* and *A. butyracea*. Analyses and figures were made in NicheA [29]

Niche overlap between the genus *Rhodnius* and infested palms represented 93.48% of the *Rhodnius* niche but only 57.38% of the infested palms niche (Fig 2a). The entire niches of *R. neglectus* and *R. pictipes* and almost all the niche volume of *R. prolixus* and *R. robustus* fell inside the niche of infested palms (Table 2).

**Table 2:** Percentage of *Rhodnius* niche volume shared with palm species

Species (niche volume)	*R. neglectus* (873)	*R. pictipes* (201)	*R. prolixus* (113)	*R. robustus* (143)
Infested palms (2098)	100	100	91.15	96.50
*Ac. aculeata* (254)	**25.60**	**76.63**	59.86	**77.56**
*As. aculeatum* (500)	33.98	92.49	75.33	**87.31**
*A. butyracea* (96)	8.80	40.33	**52.60**	**56.38**
*A. maripa* (182)	13.57	**65.98**	**64.46**	**79.38**
*A. phalerata* (239)	**25.77**	74.49	48.63	73.35
*M. flexuosa* (1624)	**92.77**	95.47	81.70	90.53
*O. bataua* (191)	13.41	**67.28**	**73.47**	**87.03**

*Notes*: Niche overlaps were normalized by each *Rhodnius* species niche volume. Niche overlap in bold are for infestations reported in the literature (see Additional file 1: Table S1)

When analyzed by species, niche volumes showed great variation; for example, the niche of *R. neglectus* was more than 7 times greater than the niche of *R. prolixus*, and the niche of *M. flexuosa* was more than 17 times greater than the niche of *A. butyracea* (Table 2). Niche overlap was found among all the *Rhodnius* and palm species compared; however, in each *Rhodnius* species, the proportion of niche overlap varied notably among palm species (Table 2). The maximum niche overlap was found between *R. pictipes* and *M. flexuosa* (95.47% of the niche of *R. pictipes*; Fig. 2b), while the minimum overlap was found between *R. neglectus* and *A. butyracea* (8.8% of the niche of *R. neglectus*; Fig 2c).

Considering palms, all of the species overlapped most of their niche volume with the genus *Rhodnius* niche (Table 3). *Attalea phalerata* and *Ac. aculeata* were the palm species sharing the highest proportions of their niches, while *M. flexuosa* was the one sharing the lowest proportion.

**Table 3:** Percentage of palms niches shared with species of the genus *Rhodnius*

Species (niche volume)	*Rhodnius* (1289)
*Ac. aculeata* (254)	92.12
*As. aculeatum* (500)	75.00
*A. butyracea* (96)	88.54
*A. maripa* (182)	79.67
*A. phalerata* (239)	94.14
*M. flexuosa* (1624)	62.19
*O. bataua* (191)	78.53

*Notes*: Niche overlaps were normalized by each palm species niche volume

**Fig. 3:**
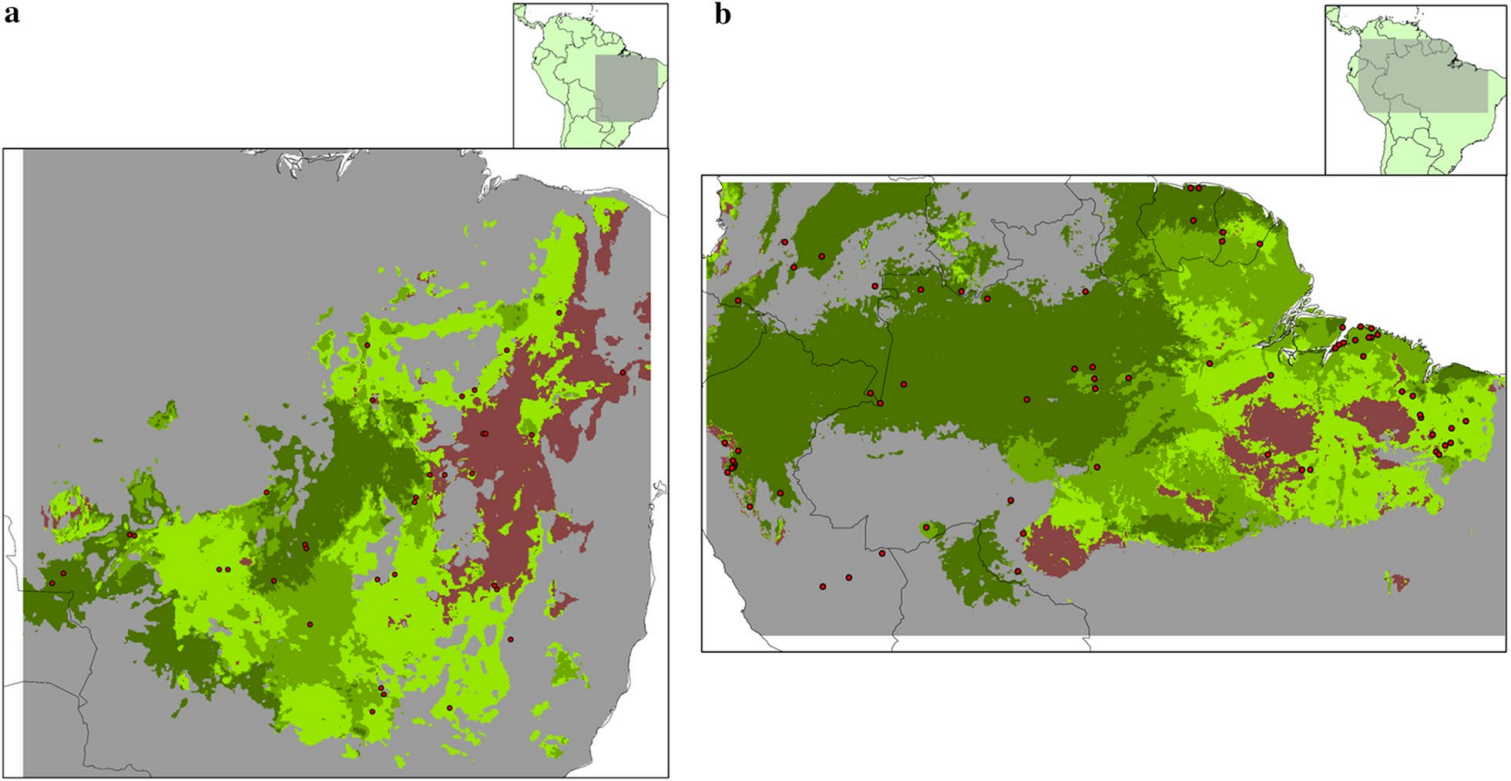
Suitable areas for palms inside the potential distributions of *Rhodnius neglectus* (**a**) and *Rhodnius pictipes* (**b**). Red points indicate *Rhodnius* species occurrences inside MVEs. Grey indicates unsuitable habitats for the *Rhodnius* species. Green and brown indicates suitable habitats for the *Rhodnius* species (light green, suitable habitats for only one palm species; intermediate green, suitable for two palm species; dark green, suitable for three or more palm species; brown, not suitable for any of the palm species modeled). Geographical extensions were based on the area covered by the occurrences. Presence area was based on a 10% training omission rate. Maps were constructed with ArcGIS 10.4

**Fig. 4:**
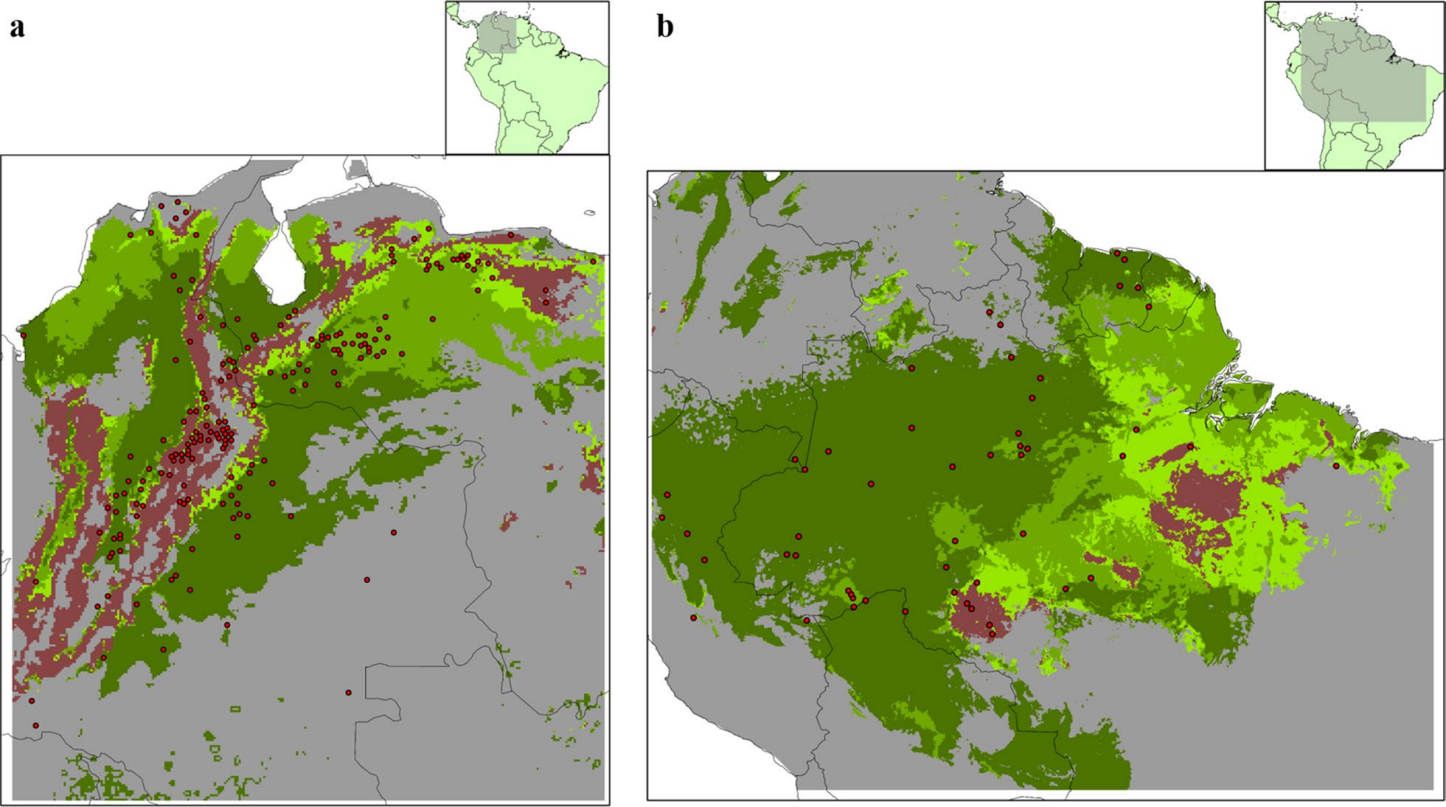
Suitable areas for palms inside the potential distributions of **a***Rhodnius prolixus* and **b***Rhodnius robustus*. Red points indicate *Rhodnius* species occurrences inside MVEs. Grey indicates unsuitable habitats for the *Rhodnius* species. Green and brown indicates suitable habitats for the *Rhodnius* species (light green, suitable habitats for only one palm species; intermediate green, suitable for two palm species; dark green, suitable for three or more palm species; brown, not suitable for any of the palm species modeled). Geographical extensions were based on the area covered by the occurrences. Presence area was based on a 10% training omission rate. Maps were constructed with ArcGIS 10.4

When palms and *Rhodnius* MVEs were projected and compared in geographical space, we found zones with suitable conditions for at least one infested palm species (from the selected palms) inside the *Rhodnius* species predicted distributions; these zones covered at least 75% of the *Rhodnius* presence area (Table 4, Figs. 3 and 4). For *R. pictipes*, *R. prolixus* and *R. robustus* distributions, a great proportion of the presence area was suitable for three or more palm species (Table 4, Figs. 3 and 4). In those *Rhodnius* species, a small proportion of the presence area was suitable to only one palm species.

**Table 4:** Percentage of palm suitable areas inside the *Rhodnius* spp. presence areas

No. of palm species	*R. neglectus*	*R. pictipes*	*R. prolixus*	*R. robustus*
0	18.78	7.46	22.52	4.57
1	43.34	19.44	8.44	13.27
2	17.56	21.91	24.55	19.43
3 or more	20.32	51.20	44.49	62.73

*Notes*: Palm areas are discriminated by the number of palm species that could be present

Almost all *Rhodnius* and palm species had areas of potential co-occurrence; nevertheless, inside each *Rhodnius* presence area the proportion of palm co-occurrence was highly variable among palm species (Table 5), corresponding with the results of niche overlap. Each *Rhodnius* species shared a high proportion of suitable areas with certain palm species, but a very low proportion with others (e.g. wide sharing of *R. prolixus* with *A. butyracea* but very small with *A. phalerata*). Considering palm species, the only palm sharing a high proportion of suitable areas with all the *Rhodnius* species was *M. flexuosa* (Table 5). For the remaining palms, the area of co-occurrence with at least one *Rhodnius* species was very small (less than 0.05).

**Table 5:** Percentage of *Rhodnius* spp. suitable areas shared with each palm species

Species	*R. neglectus*	*R. pictipes*	*R. prolixus*	*R. robustus*
*Ac. aculeata*	**65.66**	**4.39**	57.38	**9.72**
*As. aculeatum*	2.24	55.91	9.51	**57.80**
*A. butyracea*	0	31.14	**67.31**	**26.75**
*A. maripa*	0	**52.99**	**26.91**	**42.18**
*A. phalerata*	**34.08**	20.25	0.07	38.37
*M. flexuosa*	**39.20**	71.82	44.23	72.47
*O. bataua*	0	**42.82**	**45.44**	**37.91**

*Notes*: Proportions in bold font are for *Rhodnius*-palm interactions reported in the literature (see Additional file 1: Table S1)

### Palm distributions as predictors in *Rhodnius* ENMs

For all the *Rhodnius* species, ENMs run with and without palm distributions had pAUC ratios higher than the null model line (i.e. pAUC ratios > 1) (Table 6). In *R. neglectus* and *R. robustus*, pAUC ratios were significantly lower in models with palm distributions, while in *R. pictipes* and *R. prolixus*, pAUC ratios were higher in models with palms but the differences were not significant. Both omission rates (10% and 0%) were similar in all the model comparisons except in *R. prolixus* where a 0% omission rate was significantly higher in ENMs with palms (Table 7). In *R. neglectus*, *R. pictipes* and *R. prolixus*, the 0% omission presence area reduced significantly in ENMs with palms, covering less area of predicted presence with a similar omission rate. However, this pattern was not observed in 10% omission rate presence areas.

**Table 6:** Partial AUC with E = 0.10 for the *Rhodnius* ecological niche models with and without palm distributions

Statistic	Palms included	*R. neglectus*	*R. pictipes*	*R. prolixus*	*R. robustus*
pAUC value	No	0.762 ± 0.013	0.445 ± 0.046	0.772 ± 0.016	0.559 ± 0.035
	Yes	0.718 ± 0.015	0.462 ± 0.037	0.786 ± 0.014	0.521 ± 0.030
pAUC ratio	No	1.609 ± 0.018	1.269 ± 0.043	1.614 ± 0.023	1.383 ± 0.034
	Yes	1.553 ± 0.019*	1.288 ± 0.034	1.636 ± 0.021	1.301 ± 0.047*

*Significant difference between models with and without palms (paired *t*-test, significance level = 0.05)

*Notes*: All values correspond to mean ± standard error (based on 10 repetitions)

**Table 7:** Omission rates 10% and 0% for *Rhodnius* ecological niche models with and without palm distributions

Statistic	Palms included	*R. neglectus*	*R. pictipes*	*R. prolixus*	*R. robustus*
OR10%	No	0.124 ± 0.020	0.152 ± 0.222	0.123 ± 0.016	0.145 ± 0.039
	Yes	0.152 ± 0.031	0.156 ± 0.019	0.108 ± 0.010	0.160 ± 0.027
Presence area 10%	No	0.224 ± 0.007	0.380 ± 0.016	0.208 ± 0.004	0.397 ± 0.014
	Yes	0.230 ± 0.004	0.415 ± 0.013*	0.204 ± 0.002	0.426 ± 0.012
OR0%	No	0.010 ± 0.006	0.008 ± 0.005	0	0.030 ± 0.017
	Yes	0.014 ± 0.010	0.008 ± 0.005	0.014 ± 0.003*	0.055 ± 0.012
Presence area 0%	No	0.712 ± 0.035	0.946 ± 0.005	0.969 ± 0.001	0.648 ± 0.015
	Yes	0.395 ± 0.009*	0.869 ± 0.013*	0.648 ± 0.024*	0.690 ± 0.015

*Significant difference between models with and without palms (paired *t*-test, significance level = 0.05)

*Notes*: All values correspond to mean ± standard error (based on 10 repetitions)

Most of the *Rhodnius* models predicted an area of distribution adjusted to the occurrence points. No differences were observed between predictions of models with and without palms; small differences were mostly concentrated in the borders of the presence areas showing no clear pattern (Additional file 1: Figures S1–S4). Prediction uncertainty was similar in both types of models (with and without palms) (Additional file 1: Figures S1–S3) except in *R. robustus*, where it slightly increased in models with palms in several zones of the distribution (Additional file 1: Figure S4).

Finally, palm distributions showed to be a relevant predictor for the *Rhodnius* ENMs (Table 8). In *R. neglectus*, *R. prolixus* and *R. robustus*, more than one palm species showed high contributions to the models, with *Ac. aculeata* distribution a common important factor for the three *Rhodnius* species. In the ENMs without palms, NDVI and temperature were very important environmental factors highly contributing to the models of the four *Rhodnius* species.

**Table 8:** More important variables contributing to *Rhodnius* spp. ecological niche models

*Rhodnius* species	Environmental variables only	Environmental variables and palm distributions
*R. neglectus*	Mean NDVI	LST annual phase
	NDVI annual phase	*A. butyracea*
	LST annual phase	*A. aculeata*
	Precipitation of warmest quarter	*A. aculeatum*
*R. pictipes*	Mean NDVI	Mean NDVI
	NDVI annual phase	Annual precipitation
	Precipitation of warmest quarter	Precipitation of warmest quarter
	Annual precipitation	*A. phalerata*
*R. prolixus*	Mean NDVI	Mean LST
	Mean LST	NDVI annual phase
	Annual mean temperature	*A. maripa*
	Annual precipitation	*A. aculeata*
*R. robustus*	Mean NDVI	Annual mean temperature
	Mean LST	Annual precipitation
	Annual mean temperature	*A. aculeatum*
	Annual precipitation	*A. aculeata*

## Discussion

As for most ecological relations, general patterns could not be found for *Rhodnius*-palm interactions, but in terms of niche similarity, overlap was found between the genus *Rhodnius* and infested palms and among all *Rhodnius* and palm species. Almost all the environmental conditions suitable for *Rhodnius* triatomines were suitable for at least one infested palm species, while almost 40% of environmental conditions suitable for infested palms were not suitable for *Rhodnius* triatomines. This result could indicate that the *Rhodnius* niche could be somehow influenced by the palms presence, showing a possible dependence. Considering the analyses made by *Rhodnius* species, the association was even more noticeable, since the entire set of suitable conditions for *R. neglectus* and *R. pictipes* were also suitable for infested palms.

Palm and *Rhodnius* species shared, to a greater or lesser extent, environmental requirements, and the degree of niche similarity depended critically on the species involved. In the four *Rhodnius* species, at least one palm species shared almost all the suitable environmental conditions with the *Rhodnius* species (minimum overlap over 80% of the *Rhodnius* species niche). In most of the *Rhodnius*-palm interactions reported in the literature (Table 2), the overlap between niches was high (more than a half of the *Rhodnius* species niche). Only two reported *Rhodnius*-palm interactions had a relatively low niche overlap (less than 30%) constituted by *R. neglectus* with *Ac. aculeata* and with *A. phalerata*. This reduced overlap can be explained as a result of the normalization process since a niche overlap could be wide in volume, but it becomes small when compared to a huge niche. That is the case with *R. neglectus*, which had the largest niche among the *Rhodnius* species. Additionally, the proportion of niche overlap was also affected by the palm niche volume. Palm species with the largest niches such as *M. flexuosa*, had the biggest mean niche overlap with *Rhodnius* species (90.11%), while *A. butyracea*, with the smallest niche, had the minimum niche overlap (39.53%). However, all seven palm species shared a great part of their environmental requirements with at least one *Rhodnius* species (Table 3).

Regarding comparisons in geographical space, most of the areas with suitable conditions for *Rhodnius* species were also suitable for more than one palm species in the majority of the locations (Table 4, Figs. 3 and 4). These areas corresponded geographically with zones of high richness of *Rhodnius* spp. and palms in northern and central South America. The genus *Rhodnius* has shown unimodal richness distribution strongly skewed toward the low latitudes in the Northern Hemisphere [10], while palms show a great diversification in the Andean, Amazon and Central Brazilian regions [17].

From the *Rhodnius*-palm interactions reported in the literature (Additional file 1: Table S1), 12 shared relatively large suitable areas (Table 5), indicating a possible relationship between *Rhodnius*-palm co-occurrence and palm infestation. However, two observations do not support the statement. First, some *Rhodnius*-palm species combinations with wide potential co-occurrence areas had no reported infestations, and secondly, *R. pictipes* and *R. robustus* infestation was reported in *Ac. aculeata* palms [35, 36], but the areas of potential co-occurrence were very scarce (Table 5). For the first situation, it is important to mention that the palm species involved was *M. flexuosa*, which had the most extended geographical range and the widest niche. When a palm species has a very large niche and a vast presence area, it is more likely that it will include several *Rhodnius* species distributions. Therefore, palm infestation by a *Rhodnius* species is not guaranteed to occur when the palm and the *Rhodnius* species share vast suitable areas; nonetheless, co-occurrence could be an initial step for a further infestation of palms by insects. Other factors intervening at a smaller spatial scale such as palm morphology and associated fauna would be the determinants for the infestation of a particular palm [37]. It is also important to mention that previous palm infestation reports are far from being systematic studies covering great range extensions, and most of them are local studies focused on small areas compared to the huge geographical extension considered in this study [6, 38–43]. Most of the geographical areas covered by these ENMs have not been sampled yet.

Almost every *Rhodnius*-palm niche overlap in the environmental space (Table 2) was larger than that in the geographical space (Table 5), suggesting that high niche similarity was not always associated to large areas of potential co-occurrence; the only exception was *R. pictipes* and *A. butyracea*. For example, *A. aculeata* shared a very high proportion of *R. pictipes* niche volume (76.63%), but only a small proportion of suitable geographical area (9.72%), suggesting that the spatial distribution of environmental conditions is relevant to explain co-occurrence through niche similarity.

Areas with no suitable conditions for infested palms (Figs. 3 and 4) can be interpreted as not suitable for the selected seven palm species but suitable for other infested palms not included in the study (such as *A. speciosa*, *Cc. nucifera* and *Cp. tectorum*, etc.), or areas with no suitable conditions to any palm species (e.g. very elevated zones). For *R. prolixus*, no suitable areas were placed in elevated locations, along the Andean region. *Rhodnius* presence in zones with no predicted presence of the selected palms could be explained by infestations of different palm species or by ecological processes as domiciliation. *Rhodnius prolixus* in Andean locations have been observed in human dwellings [7], where insect populations can establish without the presence of palms in the proximity [44]. *Rhodnius neglectus* is considered as a synanthropic species that invades and sporadically colonize man-made ecotopes, and *R. robustus* and *R. pictipes* invade but do not colonize houses [39]. Moreover, *R. robustus* and *R. pictipes* have been found in other habitats different to palms, such as bromeliads [4].

To determine if palm presence could be a predictor of *Rhodnius* species distribution, *Rhodnius* ENMs were run with and without palm distributions as environmental variables. Performance statistics (pAUC and omission rates) were similar in both types of models. However, when palm distributions were used, they demonstrated to be relevant predictors for *Rhodnius* models compared to the environmental variables (Table 8). The most notorious impact of palm inclusion was the reduction of *Rhodnius* predicted presence area without increasing significantly the omission rates, and therefore, reducing the commission rate of the ENMs. This importance of palm distribution on *Rhodnius* ENMs would corroborate the spatial association between both organisms, which was also found with the niche comparison. *Rhodnius* presence would not be restricted to palm presence but the zones with palm presence could be more suitable for *Rhodnius* presence.

Association between palms and *Rhodnius* distribution could also be related to the fact that environmental variables such as temperature and precipitation have been shown to be important for both organisms. Triatomines and palms have shown a high sensitivity to climatic conditions. For example, temperature affects physiological and behavioral processes of triatomines such as egg production, hatching and immature development [45], and temperature and temperature seasonality have been shown to play an important role in explaining triatomine richness and distribution [46]. When considering palms, they are affected by temperature conditions due to their soft and water-rich tissues, their inability to undergo dormancy and their general lack of mechanisms to avoid or tolerate frost [47].

Although the ENMs performance was satisfactory, it is important to highlight that models are highly susceptible to the information available. The biased distribution of information (e.g. some areas intensively sampled in comparison to others) could limit the validity of the conclusions. In this study, our conclusions were based on the group of *Rhodnius* species and *Rhodnius*-infested palm species. Other cases of *Rhodnius*-palms reported interactions, such as *R. ecuadoriensis* in *Phytelephas aequatorialis* [48], *R. nasutus* in *Copernicia prunifera* [49], and *R. pallescens* in *A. butyracea* [50], were not considered here due to the low number of *Rhodnius* occurrences. It would be necessary to obtain more information about their occurrence to verify the magnitude of the patterns found in this study.

## Conclusions

Niche overlap was found between the genus *Rhodnius* and infested palms and among all *Rhodnius* and palm species. As expected, the *Rhodnius* niche appears to be more limited by the palms niche than *vice versa*, showing a possible dependence of *Rhodnius* presence on the distribution of palms. *Rhodnius* and palm species shared, to a greater or lesser extent, environmental requirements depending on the species involved. Most of the areas with suitable conditions for *Rhodnius* species were also suitable to palm species, being favorable for more than one palm species in the majority of the locations. Lastly, even though the presence of palms was relevant for *Rhodnius* ENMs, their effect did not increase model’s performance. This would be a consequence of the type of relationship between *Rhodnius* spp. and palms, where there are no clear inter-species associations and one *Rhodnius* species could inhabit more than one palm species.

## Supplementary information

**Additional file 1: Table S1.** Palm species infested by *Rhodnius* triatomines. **Table S2.** Parameters selected for ecological niche models using the AICc. **Figure S1.***Rhodnius neglectus* ENMs. **a**, **b** Final continuous maps (Mean of the continuous log-log outputs obtained from MaxEnt v.3.4.1). **c**, **d** Binary maps obtained using the 10% training percentile threshold. **e**, **f** Uncertainty maps (Standard deviation of the continuous log-log outputs). Maps were constructed with the *raster* R package. **Figure S2.***Rhodnius pictipes* ENMs. **a**, **b** Final continuous maps (Mean of the continuous log-log outputs obtained from MaxEnt v.3.4.1). **c**, **d** Binary maps obtained using the 10% training percentile threshold. **e**, **f** Uncertainty maps (Standard deviation of the continuous log-log outputs). Maps were constructed with the *raster* R package. **Figure S3.***Rhodnius prolixus* ENMs. **a**, **b** Final continuous maps (Mean of the continuous log-log outputs obtained from MaxEnt v.3.4.1). **c**, **d** Binary maps obtained using the 10% training percentile threshold. **e**, **f** Uncertainty maps (Standard deviation of the continuous log-log outputs). Maps were constructed with the *raster* R package. **Figure S4.***Rhodnius robustus* ENMs. **a**, **b** Final continuous maps (Mean of the continuous log-log outputs obtained from MaxEnt v.3.4.1). **c**, **d** Binary maps obtained using the 10% training percentile threshold. **e**, **f** Uncertainty maps (Standard deviation of the continuous log-log outputs). Maps were constructed with the *raster* R package.

## Data Availability

Data supporting the conclusions of this article are included within the article and its additional files. The datasets generated and analyzed during the present study are available from the corresponding author upon reasonable request.

## Abbreviations

ENM: ecological niche model
GBIF: Global Biodiversity Information Facility
LST: land surface temperature
NDVI: normalized difference vegetation index
MIR: middle infrared radiation
MVE: minimum-volume ellipsoids
PCA: principal components analysis
MaxEnt: maximum entropy algorithm
AICc: corrected Akaike information criterion
pAUC: partial area under the ROC curve
OR: omission rates
