# Supplementary material for: Co-occurrence or dependence? Using spatial analyses to explore the interaction between palms and Rhodnius triatomines
Source: Parasit Vectors. 2020 Apr 22;13:211. doi: 10.1186/s13071-020-04088-0 (PMC7178562; doi:10.1186/s13071-020-04088-0)

**Additional file 1: Table S1.** Palm species infested by *Rhodnius* triatomines.

| ***Rhodnius* species** | **Infested palm species** |
| --- | --- |
| *R. barretti* | *Attalea butyracea* [1], *Oenocarpus bataua* [1]*.* |
| *R. brethesi* | *Leopoldinia piassaba* [2]*.* |
| *R. colombiensis* | *A. butyracea* [3]. |
| *R. ecuadoriensis* | *Phytelephas aequatorialis* [4,5], *Elaeis guineensis* [6] |
| *R. nasutus* | *Acrocomia aculeata* [7], *Attalea speciosa* [8,9], *Copernicia prunifera* [4,9], *Mauritia flexuosa* [7,8,10], *Syagrus oleracea* [7]. |
| *R. neglectus* | *Ac. aculeata* [8], *Attalea phalerata* [8], *A. speciosa* [8], *M. flexuosa* [11], *Sy. oleracea* [7,8]. |
| *R. neivai* | *Copernicia tectorum* [12] |
| *R. pallescens* | *A. butyracea*[13–16], *Cocos nucifera* [14], *Cp. tectorum* [14], *Elaeis oleifera* [14,17], *O. bataua* [17]. |
| *R. pictipes* | *Ac. aculeata* [18], *Astrocaryum murumuru* [18], *Attalea maripa* [18], *O. bataua* [18]. |
| *R. prolixus* | *Ac. aculeata* [19], *A. butyracea* [20–22], *A. maripa* [20], *Cc. nucifera* [23], *Cp. tectorum* [24], *E. guineensis* [25,26], *O. bataua* [19], *Sabal mauritiiformis* [19], *Syagrus orinocensis* [19]. |
| *R. robustus* | *Ac. aculeata* [27,28], *Astrocaryum aculeatum* [29], *As. murumuru* [18], *A. butyracea* [30], *A. maripa* [18,29], *A. phalerata* [29], *A. speciosa* [29]. |
| *R. stali* | *A. phalerata* [31] |

**References**

1. Abad-Franch F, Pavan MG, Jaramillo-O N, Palomeque FS, Dale C, Chaverra D, et al. *Rhodnius barretti*, a new species of triatominae (Hemiptera: Reduviidae) from western Amazonia. Mem Inst Oswaldo Cruz. 2013;108:92–9.

2. Rocha D da S, dos Santos CM, Cunha V, Jurberg J, Galvão C. [Life cycle of *Rhodnius brethesi* Matta, 1919 (Hemiptera, Reduviidae, Triatominae), a potential vector of Chagas disease in the Amazon region]. Mem Inst Oswaldo Cruz. 2004;99:591–5.

3. Arévalo A, Carranza JC, Guhl F, Clavijo JA, Vallejo GA. Comparación del ciclo de vida de *Rhodnius colombiensis* Moreno, Jurberg & Galvão, 1999 y *Rhodnius prolixus* Stal, 1872 (Hemiptera, Reduviidae, Triatominae) en condiciones de laboratorio. Biomédica. 2007;27:119–29.

4. Noireau F, Abad-Franch F, Valente SAS, Dias-Lima A, Lopes CM, Cunha V, et al. Trapping Triatominae in Silvatic Habitats. Memias Do Inst Oswaldo Cruz. 2002;97:61–3.

5. Abad-Franch F, Palomeque FS, Aguilar VHM, Miles MA. Field ecology of sylvatic *Rhodnius* populations (Heteroptera, Triatominae): risk factors for palm tree infestation in western Ecuador. Trop Med Int Heal. 2005;10:1258–66.

6. Grijalva MJ, Palomeque FS, Villacís AG, Black CL, Arcos-Terán L. Absence of domestic triatomine colonies in an area of the coastal region of Ecuador where Chagas disease is endemic. Mem Inst Oswaldo Cruz. 2010;105:677–81.

7. Dias FBS, Bezerra CM, De Menezes Machado EM, Casanova C, Diotaiuti L. Ecological aspects of *Rhodnius nasutus* Stål, 1859 (Hemiptera: Reduviidae: Triatominae) in palms of the Chapada do Araripe in Ceará, Brazil. Mem Inst Oswaldo Cruz. 2008;103:824–30.

8. Abad-Franch F, Monteiro FA, Jaramillo O. N, Gurgel-Gonçalves R, Dias FBS, Diotaiuti L. Ecology, evolution, and the long-term surveillance of vector-borne Chagas disease: A multi-scale appraisal of the tribe Rhodniini (Triatominae). Acta Trop. 2009;110:159–77.

9. Dias FBS, de Paula AS, Belisário CJ, Lorenzo MG, Bezerra CM, Harry M, et al. Influence of the palm tree species on the variability of *Rhodnius nasutus* Stål, 1859 (Hemiptera, Reduviidae, Triatominae). Infect Genet Evol. 2011;11:869–77.

10. Gurgel-Gonçalves R, Cuba C a. C. Estrutura de populações de *Rhodnius neglectus* Lent e *Psammolestes tertius* Lent & Jurberg (Hemiptera, Reduviidae) em ninhos de pássaros (Furnariidae) presentes na palmeira *Mauritia flexuosa* no Distrito Federal, Brasil. Rev Bras Zool. 2007;24:157–63.

11. Gurgel-Gonçalves R, Duarte MA, Ramalho ED, Torre Palma AR, Romaña CA, Cuba-cuba CA. Distribuição espacial de populações de triatomíneos (Hemiptera: Reduviidae) em palmeiras da espécie *Mauritia flexuosa* no Distrito Federal, Brasil. Rev Soc Bras Med Trop. 2004;37:241–7.

12. Carcavallo R, Tonn R, Jiménez J. Notas sobre la biología, ecología y distribución geográfica de *Rhodnius neivai* Lent, 1953 (Hemiptera: Reduviidae). Bol Dir Malariol San Amb. 1976;16:169–71.

13. Pizarro Novoa JC, Romaña C. Variación estacional de una población silvestre de *Rhodnius pallescens* Barber 1932 (Heteroptera: Triatomiane) en la costa caribe colombiana. Bull Inst Fr Études Andin. 1998;27:309–25.

14. Romaña CA, Pizarro JC, Rodas E, Guilbert E. Palm trees as ecological indicators of risk areas for Chagas disease. Trans R Soc Trop Med Hyg. 1999;93:594–5.

15. Gottdenker NL, Calzada JE, Saldaña A, Carroll CR. Association of anthropogenic land use change and increased abundance of the Chagas disease vector *Rhodnius pallescens* in a rural landscape of Panama. Am J Trop Med Hyg. 2011;84:70–7.

16. Cantillo-Barraza O, Chaverra D, Marcet P, Arboleda-Sánchez S, Triana-Chávez O. *Trypanosoma cruzi* transmission in a Colombian Caribbean region suggests that secondary vectors play an important epidemiological role. Parasit Vectors. 2014;7:381.

17. Jaramillo N, Schofield CJ, Gorla D, Caro-Riaño H, Moreno J, Mejia E, et al. The Role of *Rhodnius Pallescens* as a Vector of Chagas Disease in Colombia and Panama. Res Rev Parasitol. 2000;60:75–82.

18. Ricardo-Silva AH, Lopes CM, Ramos LB, Marques WA, Mello CB, Duarte R, et al. Correlation between populations of *Rhodnius* and presence of palm trees as risk factors for the emergence of Chagas disease in Amazon region, Brazil. Acta Trop. 2012;123:217–23.

19. Fitzpatrick SO. The analysis of the relationship between domestic and silvatic populations of *Rhodnius prolixus* (Hemiptera : Reduviidae) in Venezuela by geometric morphometric and molecular methods. London School of Hygiene & Tropical Medicine; 2007.

20. D’Alessandro A, Barreto P, Saravia N, Barreto M. Epidemiology of *Trypanosoma cruzi* in the Oriental Plains of Colombia. Am J Trop Med Hyg. 1984;33:1084–95.

21. Angulo VM, Esteban L, Luna KP. *Attalea butyracea* próximas a las viviendas como posible fuente de infestación domiciliaria por *Rhodnius prolixus* (Hemiptera: Reduviidae) en los Llanos Orientales de Colombia. Biomédica. 2012;32:277–85.

22. Urbano P, Poveda C, Molina J. Effect of the physiognomy of *Attalea butyracea* (Arecoideae) on population density and age distribution of *Rhodnius prolixus* (Triatominae). Parasit Vectors. 2015;8:1–12.

23. Morocoima A, Chique J, Zavala-Jaspe R, Díaz-Bello Z, Ferrer E, Urdaneta-Morales S, et al. Commercial coconut palm as an ecotope of Chagas disease vectors in north-eastern Venezuela. J Vector Borne Dis. 2010;47:76–84.

24. Abad-Franch F, Lima MM, Sarquis O, Gurgel-Gonçalves R, Sánchez-Martín M, Calzada J, et al. On palms, bugs, and Chagas disease in the Americas. Acta Trop. 2015;151:126–41.

25. Guhl F, Pinto N, Marín D, Herrera C, Aguilera G, Naranjo JM, et al. Primer reporte de *Rhodnius prolixus* Stal, en *Elaeis guineensis* variedad Papúa, en plantaciones agroindustriales de Villanueva, Casanare. Biomedica. 2005;25:158–9.

26. Erazo D, Gottdenker NL, González C, Guhl F, Cuellar M, Kieran TJ, et al. Generalist host species drive *Trypanosoma cruzi* vector infection in oil palm plantations in the Orinoco region, Colombia. Parasit Vectors. 2019;12:274.

27. Longa ANA, Scorza V. *Acrocomia aculeata* (Palmae), hábitat silvestre de *Rhodnius robustus* en el Estado Trujillo, Venezuela. Parasitol Latinoam. 2005;60:17–24.

28. Longa A, Scorza JV. Migración de *Rhodnius robustus* (Hemiptera: Triatominae) desde *Acrocomia aculeata* (Palmae) hacia domicilios rurales en Venezuela. Boletín Malariol y Salud Ambient. 2007;47:213–20.

29. Dias FBS, Quartier M, Diotaiuti L, Mejía G, Harry M, Lima ACL, et al. Ecology of *Rhodnius robustus* Larrousse, 1927 (Hemiptera, Reduviidae, Triatominae) in *Attalea* palm trees of the Tapajós River Region (Pará State, Brazilian Amazon). Parasit Vectors. 2014;7:154.

30. Feliciangeli MD, Dujardin JP, Bastrenta B, Mazzarri M, Villegas J, Flores M, et al. Is *Rhodnius robustus* (hemiptera: Reduviidae) responsible for chagas disease transmission in western venezuela? Trop Med Int Heal. 2002;7:280–7.

31. Justi SA, Noireau F, Cortez MR, Monteiro FA. Infestation of peridomestic *Attalea phalerata* palms by *Rhodnius stali*, a vector of *Trypanosoma cruzi* in the Alto Beni, Bolivia. Trop Med Int Heal. 2010;15:727–32.

**Additional file 1: Table S2.** Parameters selected for ecological niche models using the AICc.

| ***Rhodnius* species** | **Palms included** | **Features** | **Regularization parameter** |
| --- | --- | --- | --- |
| *R. neglectus* | No | Linear and quadratic | 0.02 |
|  | Yes | Linear | 0.02 |
| *R. pictipes* | No | Linear, quadratic and product | 0.02 |
|  | Yes | Linear and quadratic | 1 |
| *R. prolixus* | No | Linear, quadratic and product | 1 |
|  | Yes | Linear, quadratic and product | 0.46 |
| *R. robustus* | No | Linear and quadratic | 0.46 |
|  | Yes | Linear and quadratic | 0.46 |

**Additional file 1: Figure S1.** *Rhodnius neglectus* ENMs. **a**, **b** Final continuous maps (Mean of the continuous log-log outputs obtained from MaxEnt v.3.4.1). **c**, **d** Binary maps obtained using the 10% training percentile threshold. **e**, **f** Uncertainty maps (Standard deviation of the continuous log-log outputs). Maps were constructed with the *raster* R package.

**a**


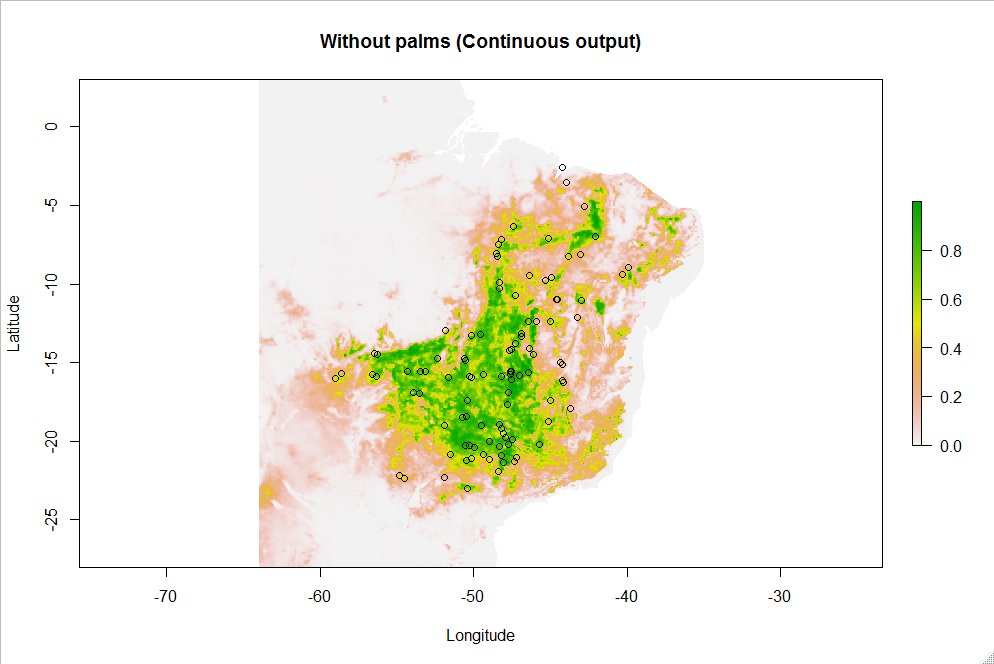


**b**


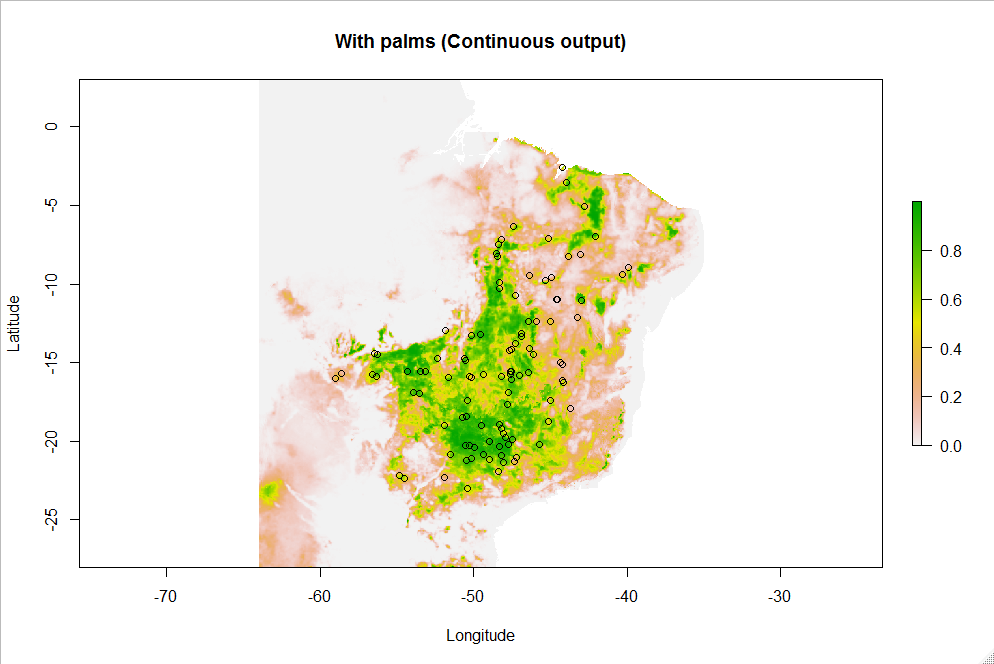


**c**


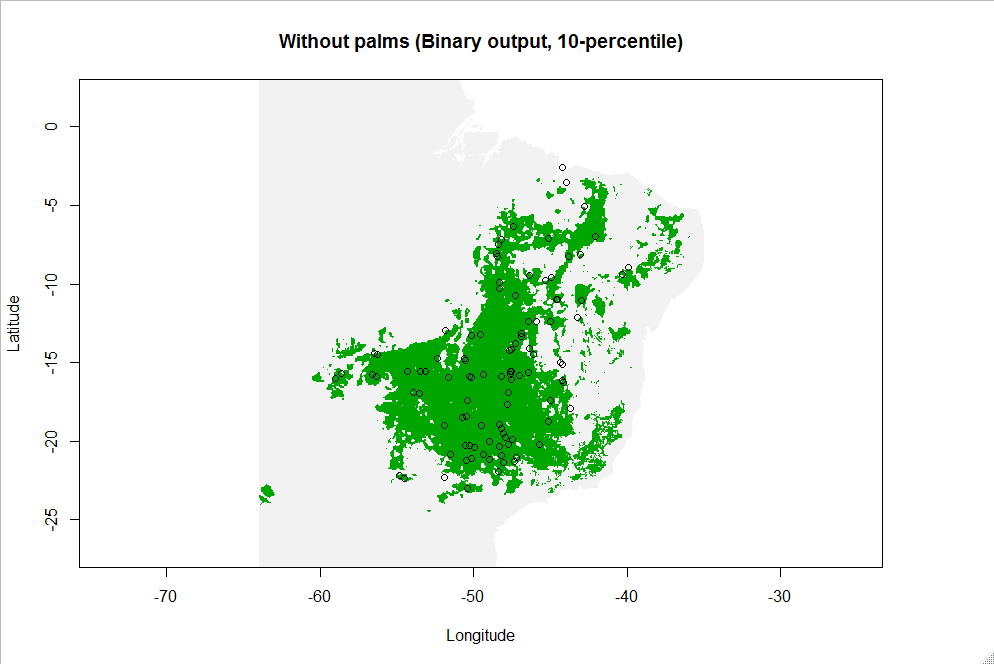


**d**


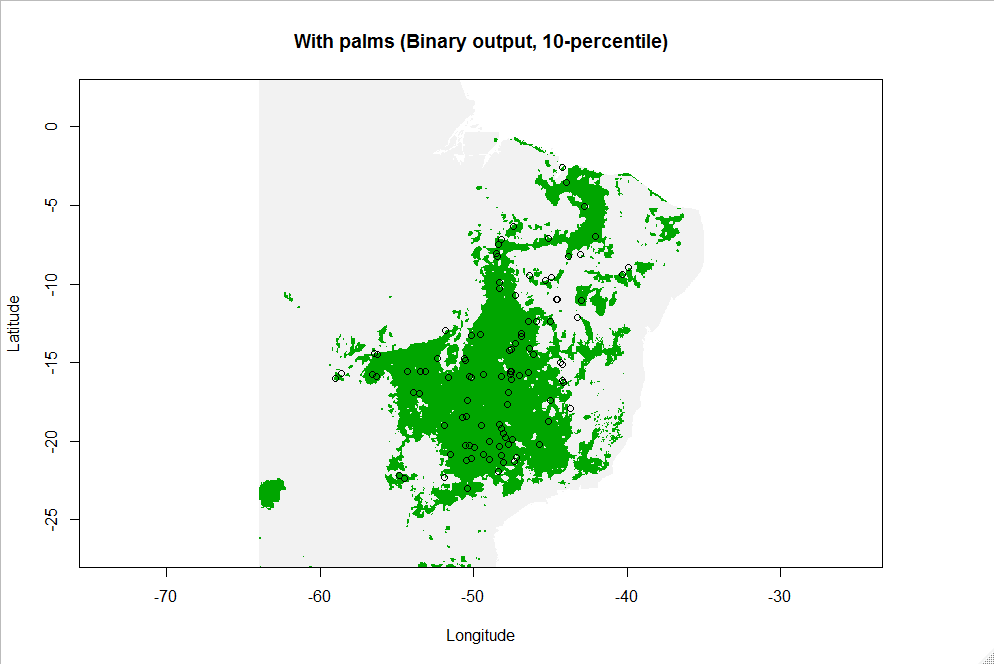


**e**


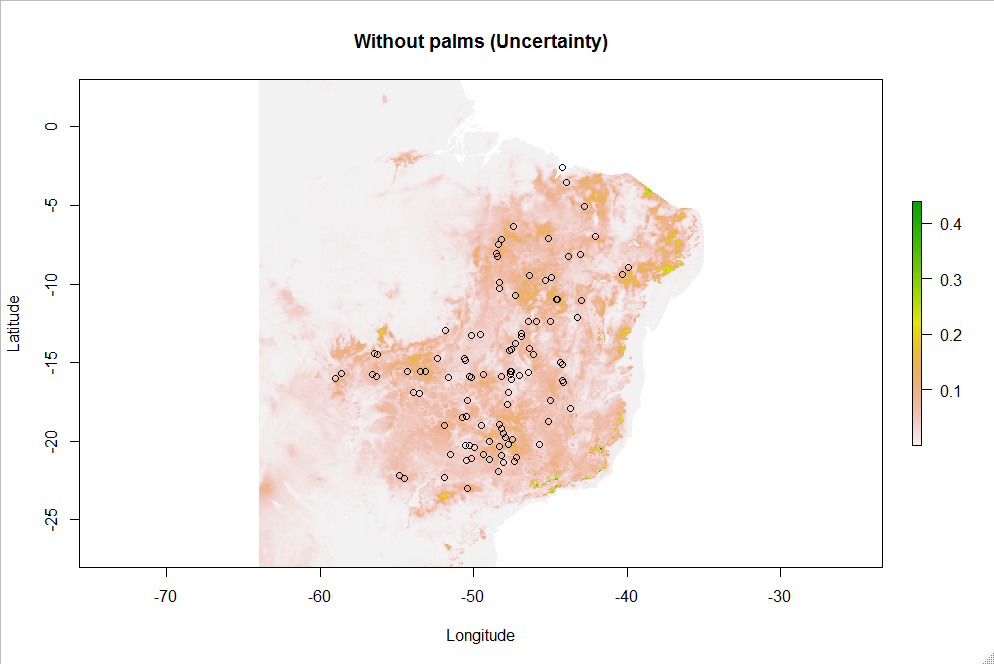


**f**


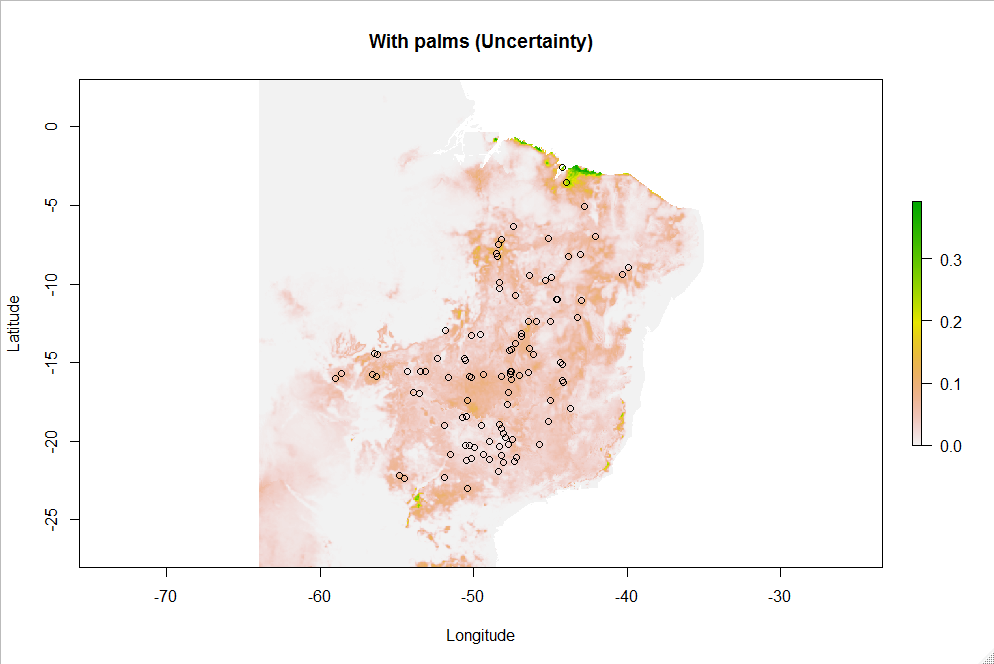


**Additional file 1: Figure S2.** *Rhodnius pictipes* ENMs. **a**, **b** Final continuous maps (Mean of the continuous log-log outputs obtained from MaxEnt v.3.4.1). **c**, **d** Binary maps obtained using the 10% training percentile threshold. **e**, **f** Uncertainty maps (Standard deviation of the continuous log-log outputs). Maps were constructed with the *raster* R package.

**a**


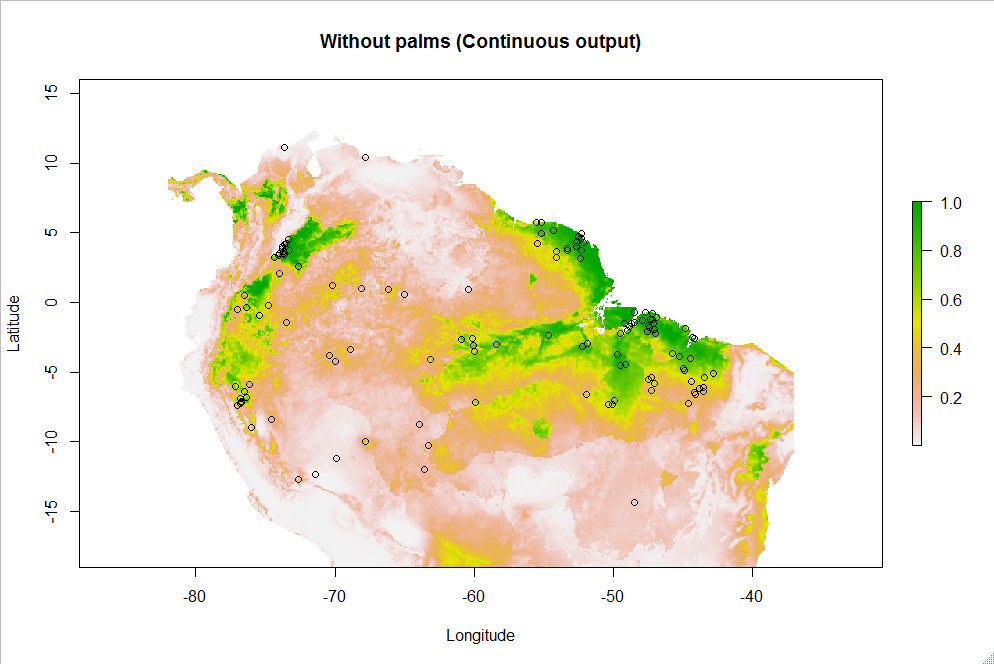


**b**


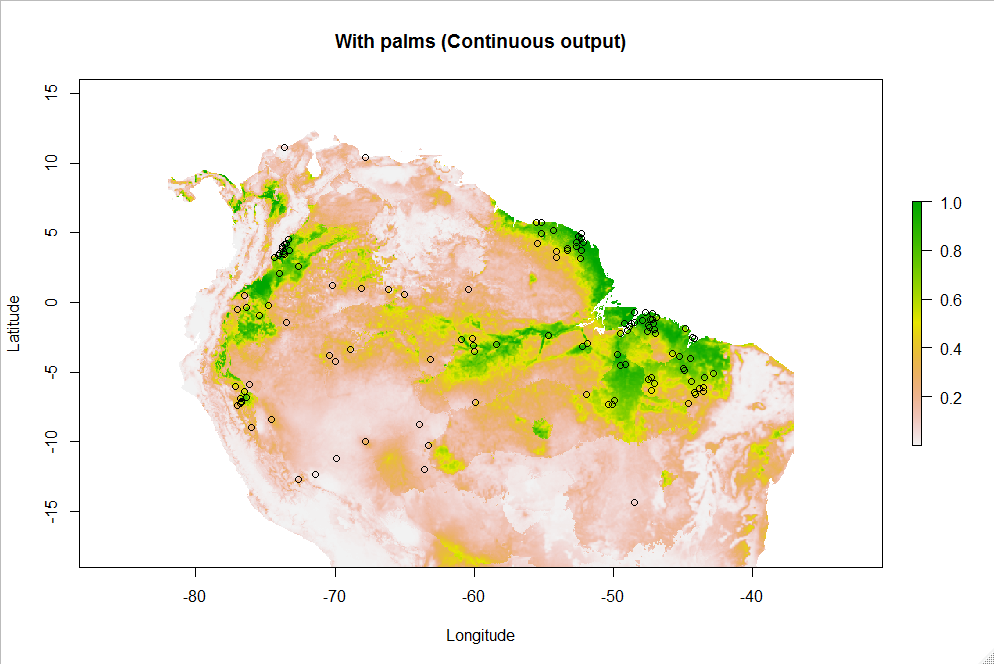


**c**


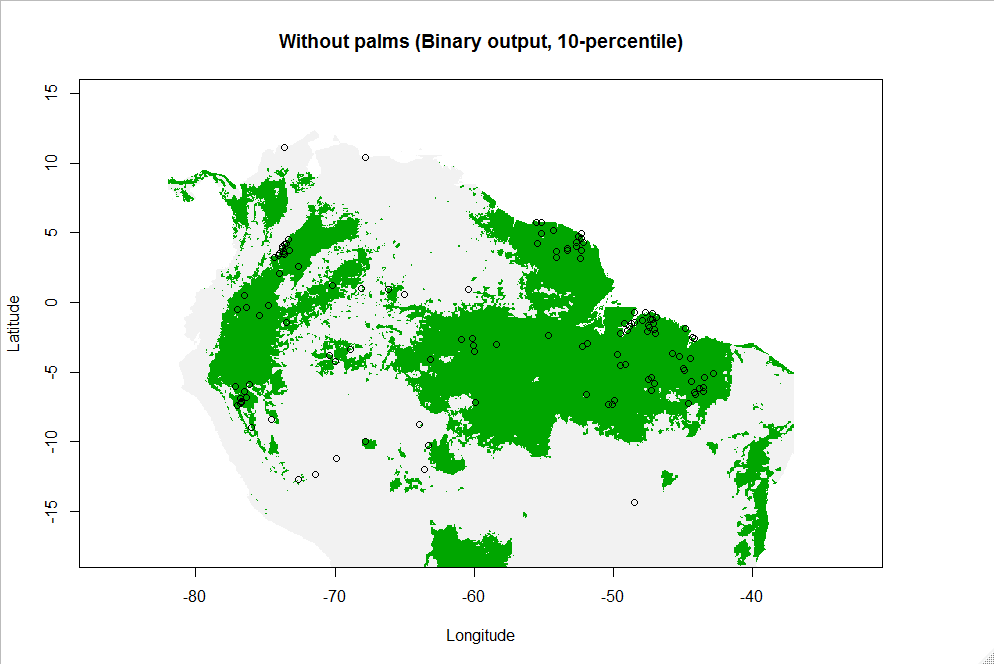


**d**


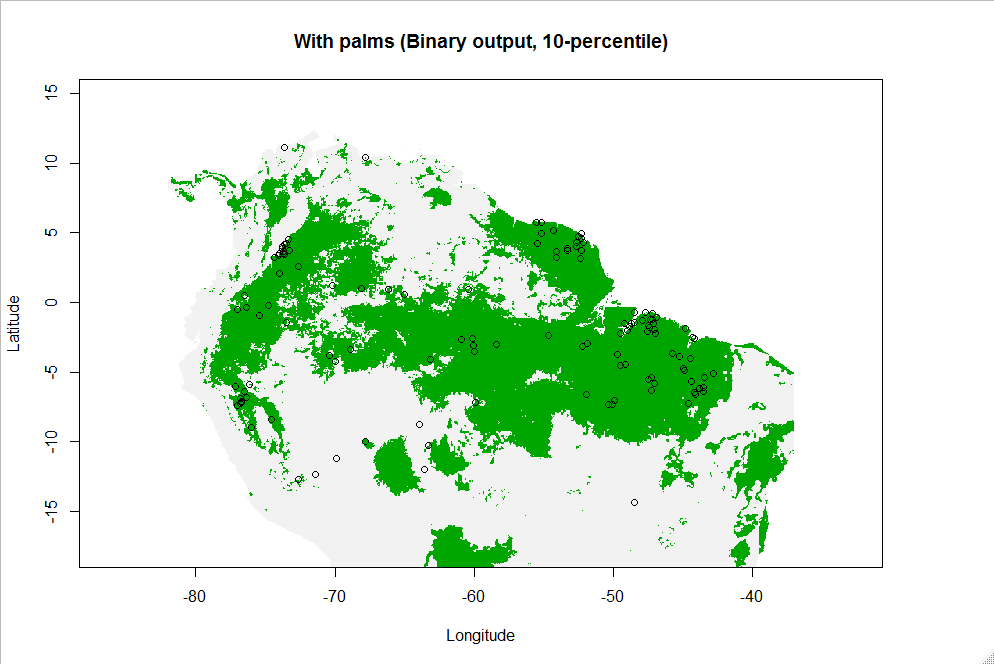


**e**


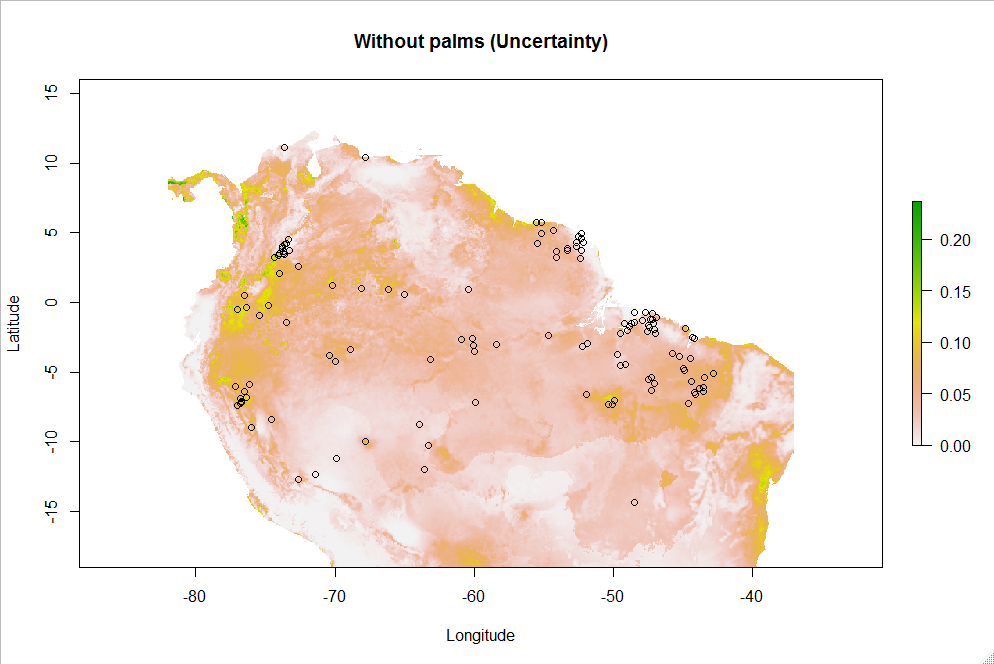

**f**


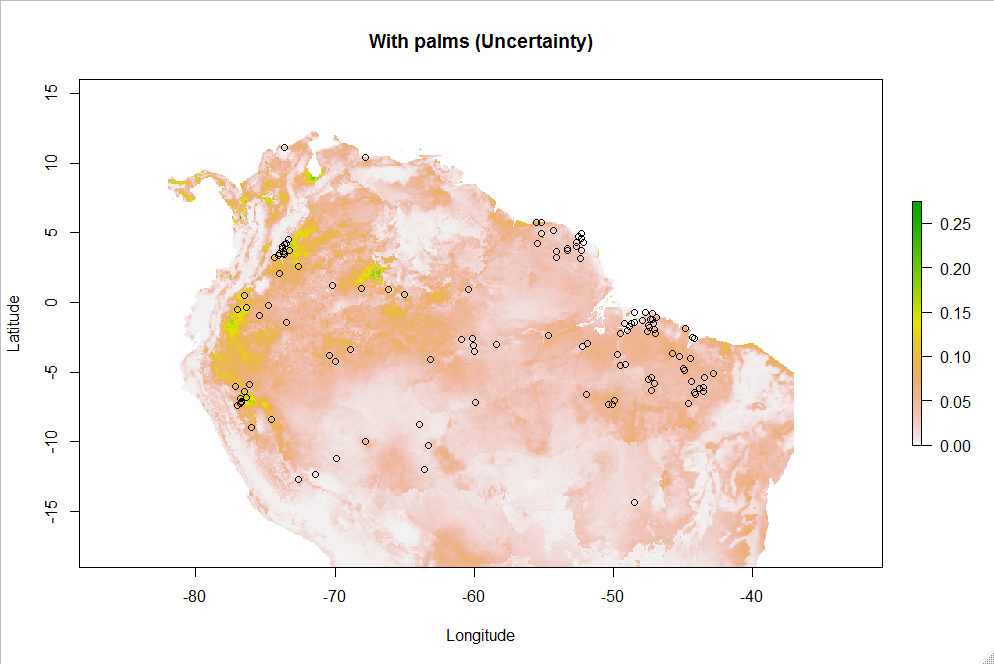


**Additional file 1: Figure S3.** *Rhodnius prolixus* ENMs. **a**, **b** Final continuous maps (Mean of the continuous log-log outputs obtained from MaxEnt v.3.4.1). **c**, **d** Binary maps obtained using the 10% training percentile threshold. **e**, **f** Uncertainty maps (Standard deviation of the continuous log-log outputs). Maps were constructed with the *raster* R package.

**a**


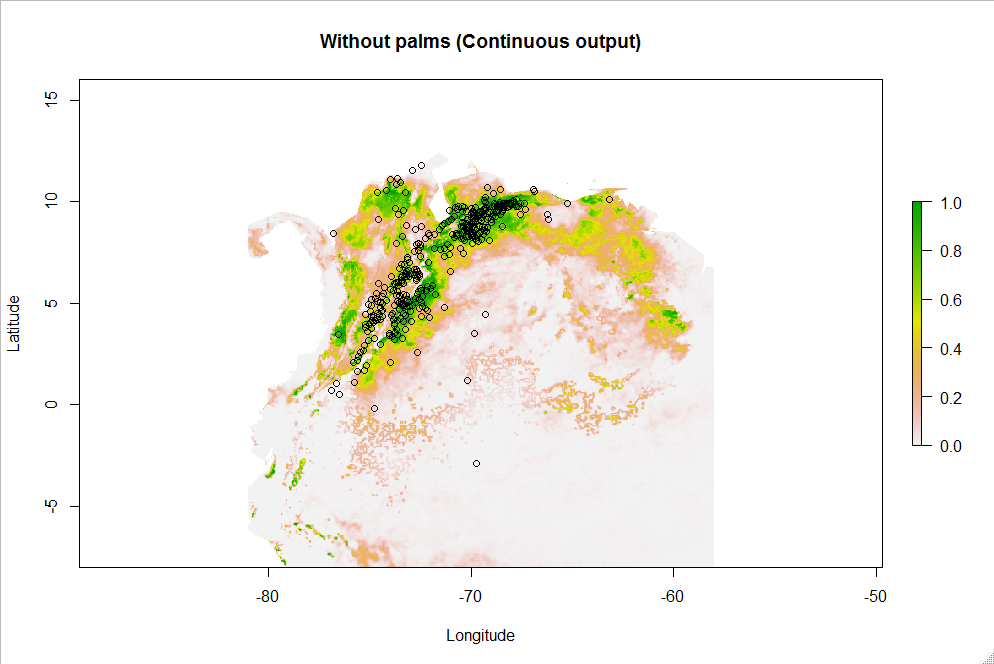


**b**


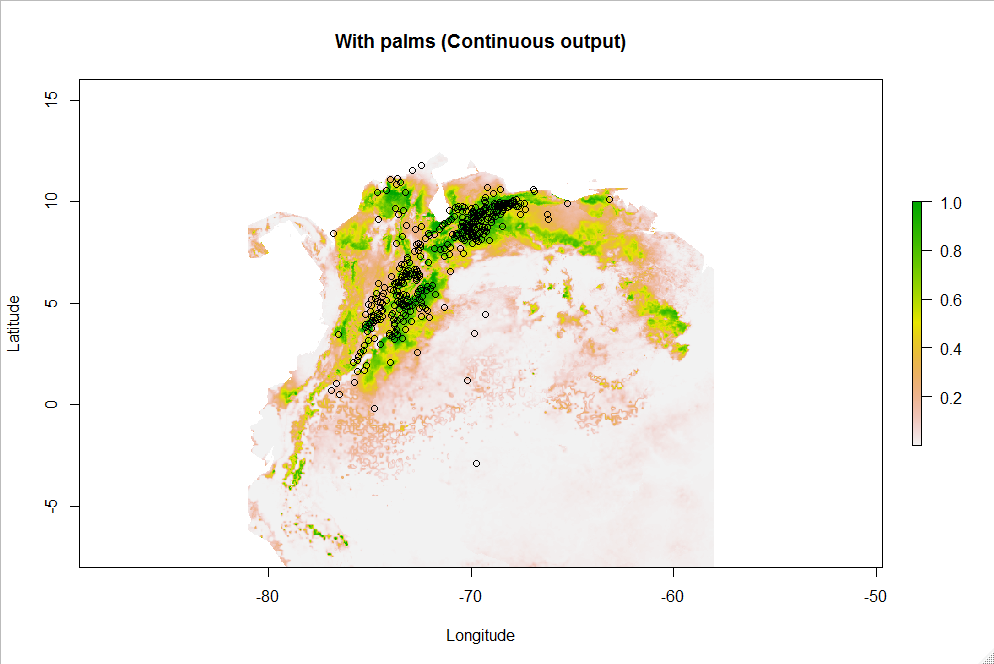


**c**


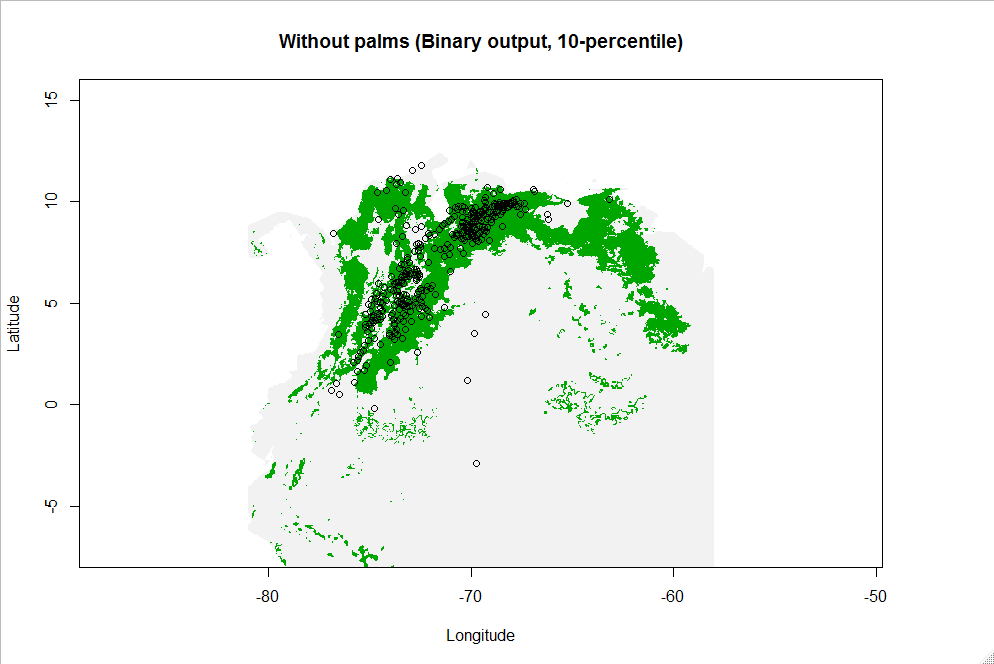


**d**


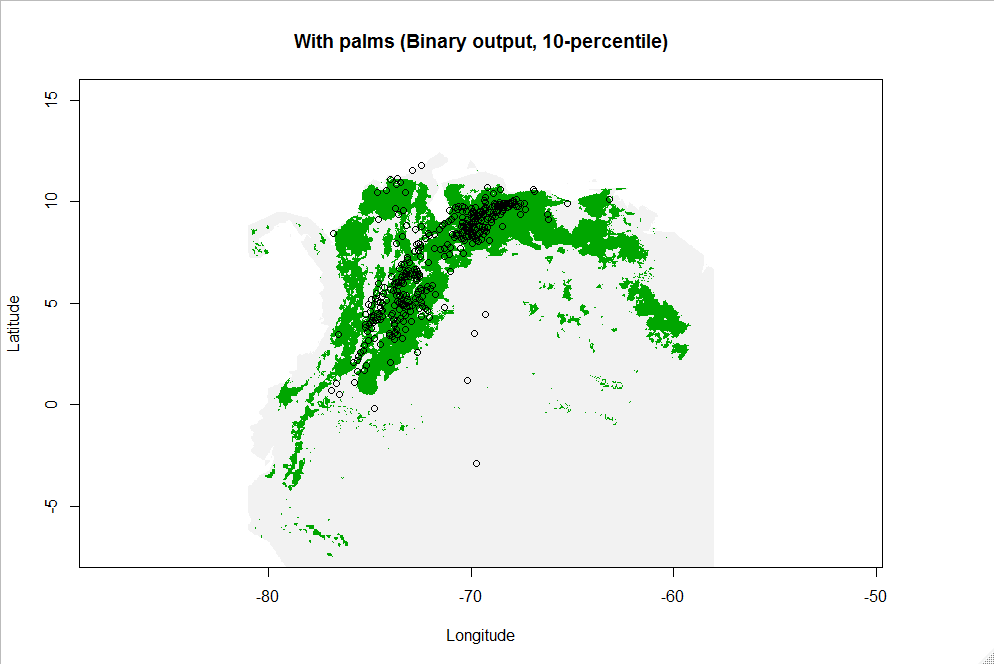


**e**


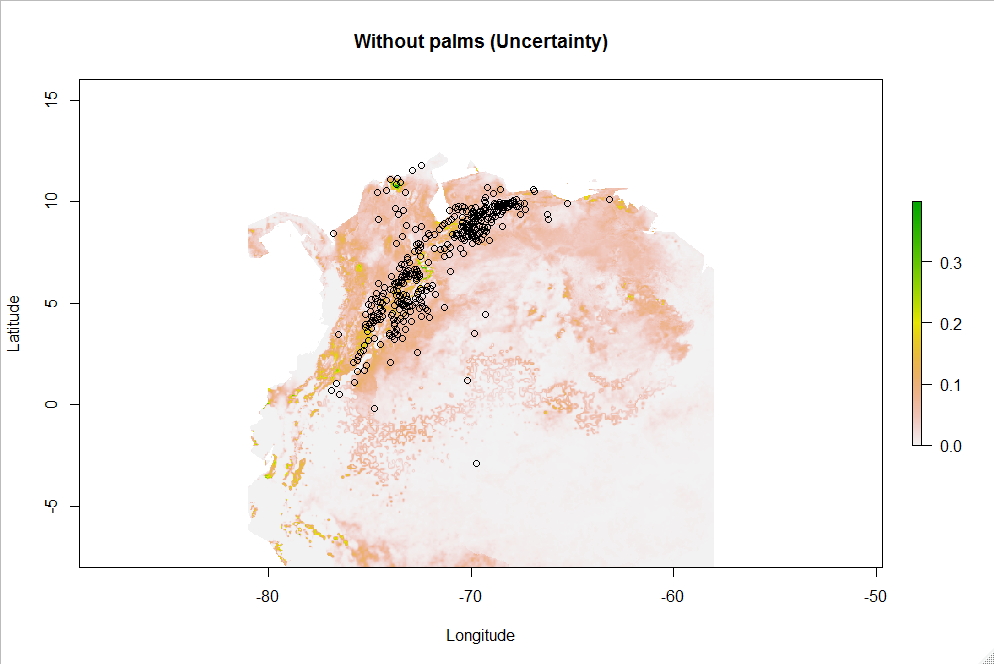


**f**


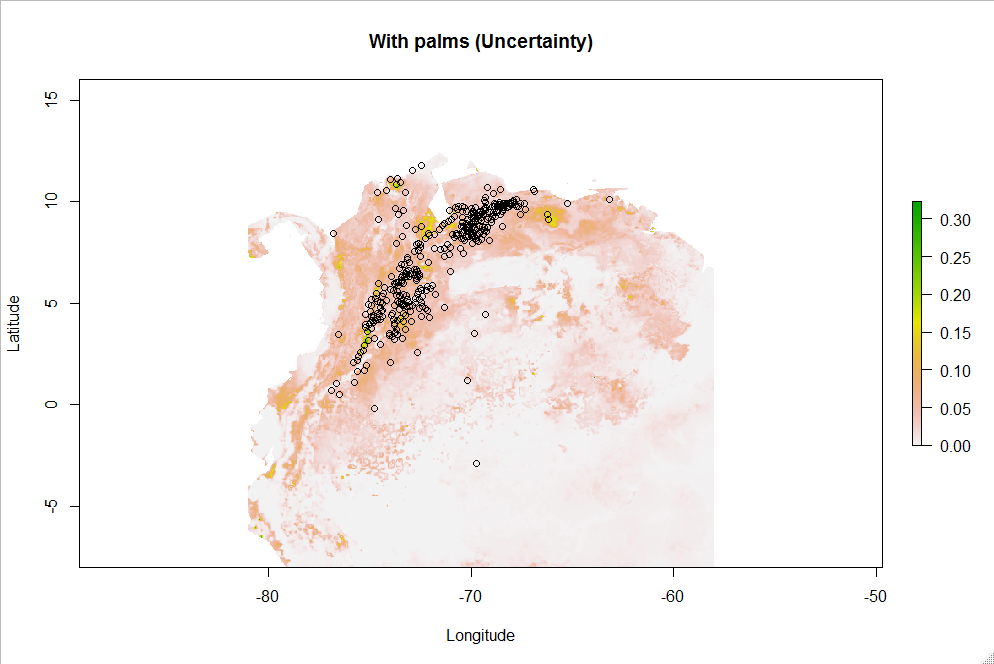


**Additional file 1: Figure S4. Figure S4.** *Rhodnius robustus* ENMs. **a**, **b** Final continuous maps (Mean of the continuous log-log outputs obtained from MaxEnt v.3.4.1). **c**, **d** Binary maps obtained using the 10% training percentile threshold. **e**, **f** Uncertainty maps (Standard deviation of the continuous log-log outputs). Maps were constructed with the *raster* R package.

**a**


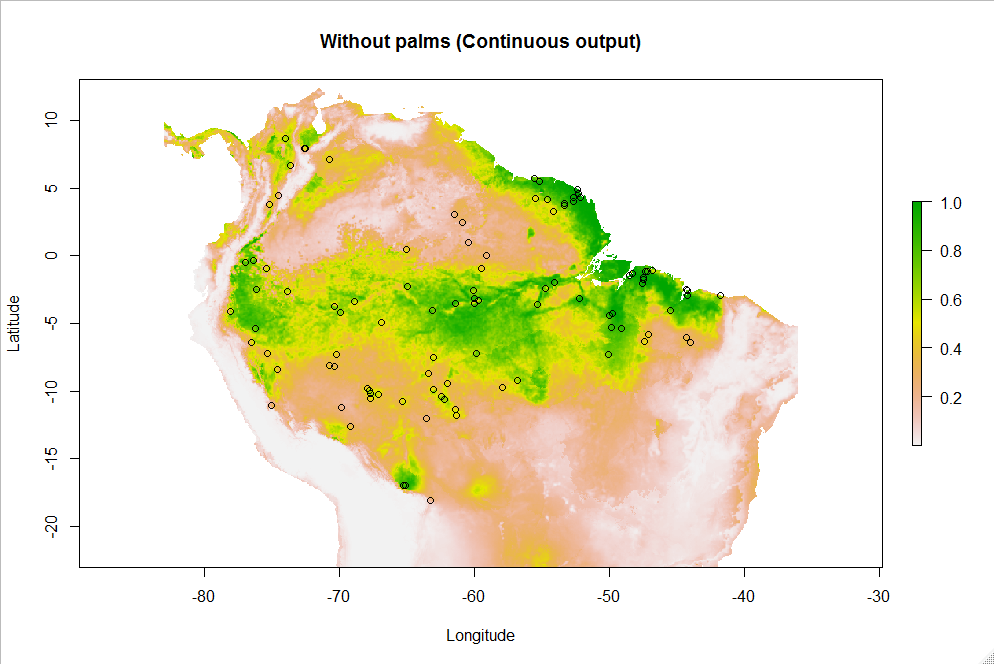


**b**


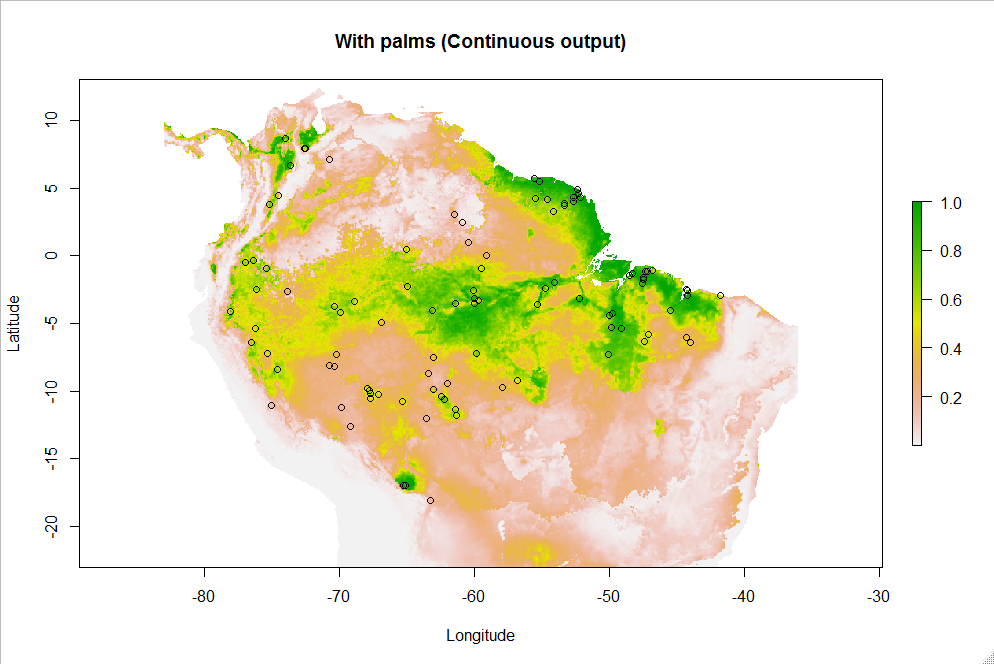


**c**


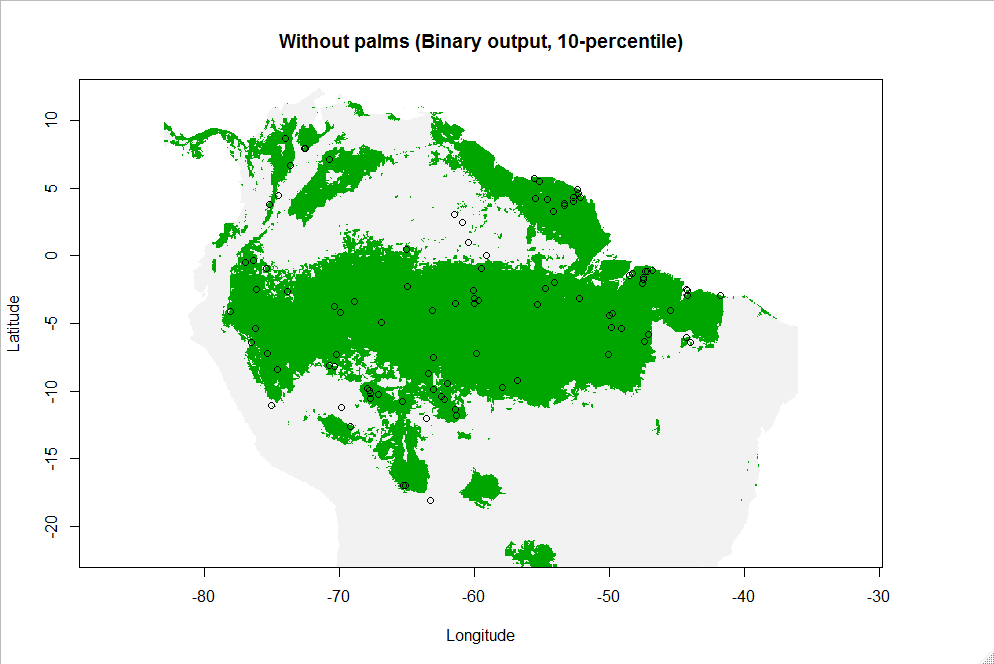


**d**


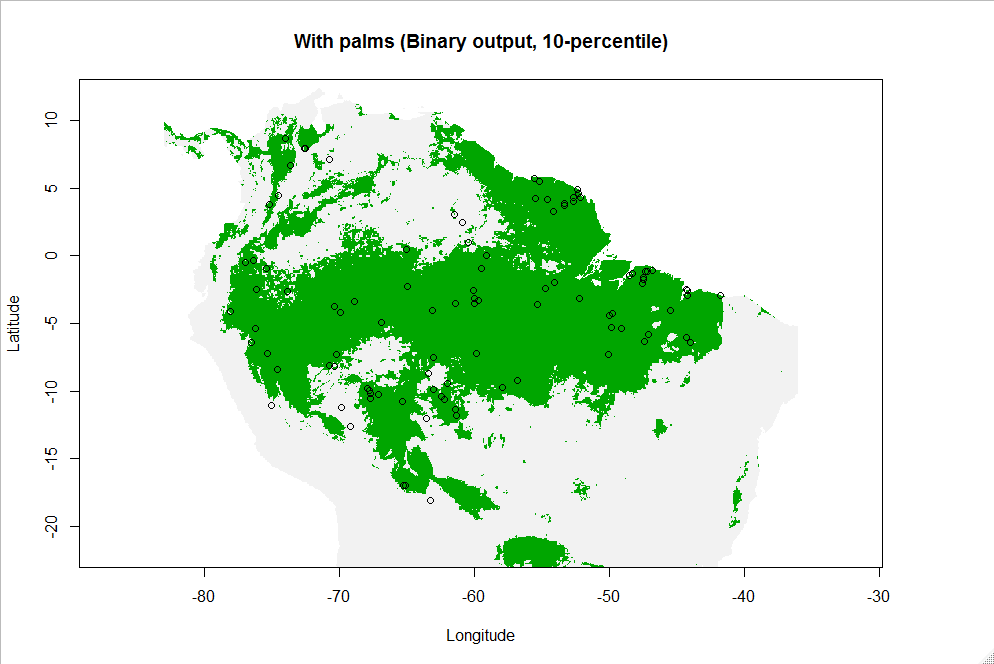


**e**


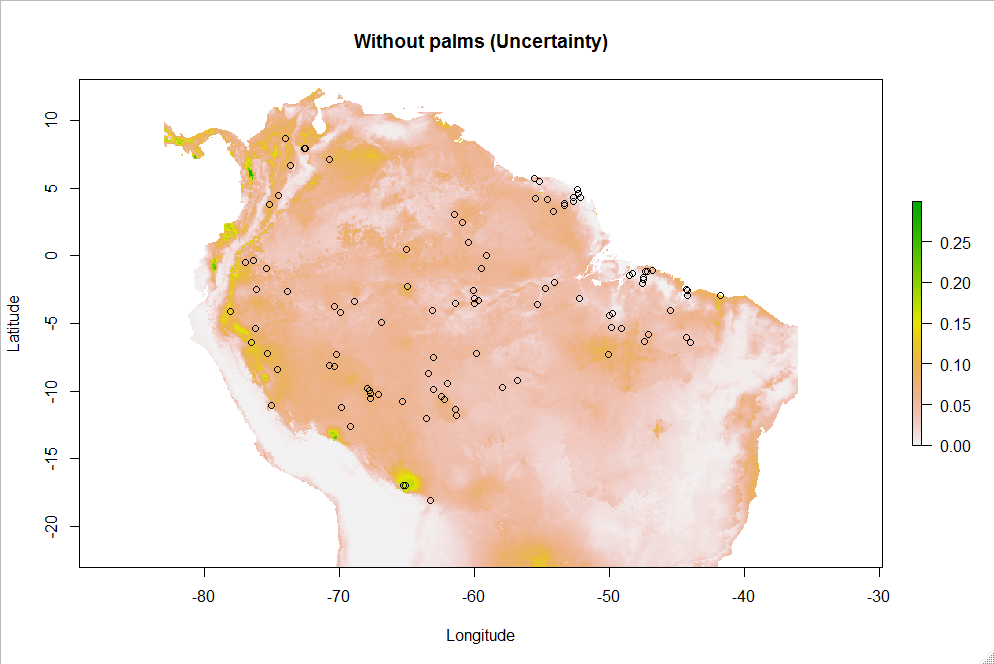


**f**


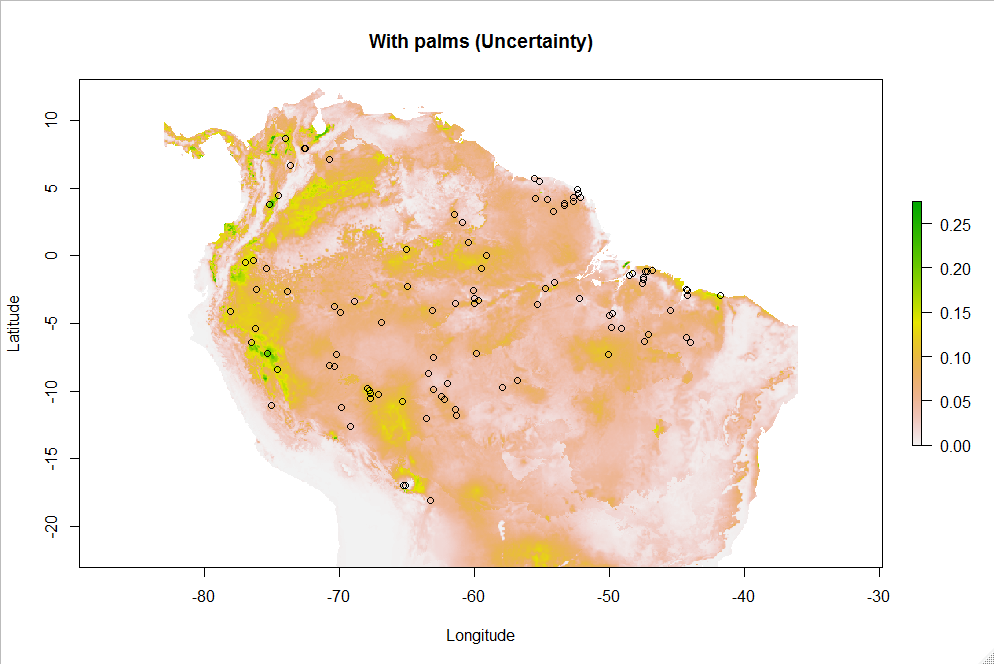

Supplement: Supplementary file 1 — Additional file 1: Table S1. Palm species infested by Rhodnius triatomines. Table S2. Parameters selected for ecological niche models using the AICc. Figure S1.Rhodnius neglectus ENMs. a, b Final continuous maps (Mean of the continuous log-log outputs obtained from MaxEnt v.3.4.1). c, d Binary maps obtained using the 10% training percentile threshold. e, f Uncertainty maps (Standard deviation of the continuous log-log outputs). Maps were constructed with the raster R package. Figure S2.Rhodnius pictipes ENMs. a, b Final continuous maps (Mean of the continuous log-log outputs obtained from MaxEnt v.3.4.1). c, d Binary maps obtained using the 10% training percentile threshold. e, f Uncertainty maps (Standard deviation of the continuous log-log outputs). Maps were constructed with the raster R package. Figure S3.Rhodnius prolixus ENMs. a, b Final continuous maps (Mean of the continuous log-log outputs obtained from MaxEnt v.3.4.1). c, d Binary maps obtained using the 10% training percentile threshold. e, f Uncertainty maps (Standard deviation of the continuous log-log outputs). Maps were constructed with the raster R package. Figure S4.Rhodnius robustus ENMs. a, b Final continuous maps (Mean of the continuous log-log outputs obtained from MaxEnt v.3.4.1). c, d Binary maps obtained using the 10% training percentile threshold. e, f Uncertainty maps (Standard deviation of the continuous log-log outputs). Maps were constructed with the raster R package. [file 13071_2020_4088_MOESM1_ESM.docx]
